# Supplementary material for: The complex swarming dynamics of malaria mosquitoes emerge from simple minimally-interactive behavioral rules
Source: PLoS Comput Biol. 2026 Aug 26;22(8):e1014685. doi: 10.1371/journal.pcbi.1014685 (PMC13529247; doi:10.1371/journal.pcbi.1014685)
Supplement: Supplementary file 1 [file pcbi.1014685.s010.pdf]

Cribellier et al. use a state-of-the-art tracking system to study mosquito swarming behavior. The premise of the paper is that this behavior can be explained by simple rules, similar to those found in boid or zonal SPP models, which are commonly used to model collective motion. Crucially, the paper proposes a discrepancy: the authors argue that such simple rules are environmentally driven rather than resulting from interactions within the group. The manuscript begins with a description of the experimental paradigm and observed collective behavior (a concise version of the behavior-focused bioRxiv submission by Poda et al.), followed by model fitting and an exploration of the different models for this behavior.

Lekking and Comparisons to Other Collectives

Mosquito swarming is essentially a form of lekking, a mating behavior observed in many animals, in which males aggregate in a defined space. The advantage of leks is that such aggregates are stable in space and time, allowing females to join. Such stability can increase overall mating success through higher aggregate density, female mate choice, and male–male competition. In this regard, at least categorically, lek mating differs from the collective behaviors mentioned by the authors, such as fish schools and bird flocks, which represent the directed motion of groups that rely on conspecific interactions. Therefore, a direct comparison between two categories of collective behavior may risk overstating the similarities.

In particular, the attempt to generalize mosquito lekking to insect collective motion more broadly, and to suggest that insect swarms are fundamentally distinct because they rely primarily on environmental cues, seems open to question. Other insect collectives, such as eusocial insects or locusts, exhibit definitely directed collective motion across considerable distances (thus less likely to be bounded by environmental clues). There, directionality indeed arises from social cues, such as pheromones; social rules govern the behavior, such as conspecific pursuit. Instead of lek mating, such insect swarms provide more suitable basis for comparison with the vertebrate collectives mentioned above.

I would therefore recommend substantial reconsideration regarding the extent to which mosquito swarming can be taken as case.

Adaptation of Boid Models

By invoking fish schooling and bird flocking, the authors adapt boid models to mosquito swarming behavior. Boid (and related zonal) models are based on differential weighting (or zones) of avoidance, alignment, and attraction, in order of increasing distance from the focal individual. Classically, all three rules involve instantaneous adjustment to neighbors' behavior. In this manuscript, only repulsion is defined in that classical sense.

1. **Attraction** is described in Figure 4 as “toward the swarm center” and in the Discussion as “the swarming mosquitoes are attracted towards a vertical center line above the visual ground marker.” The observation that mosquitoes turn at a specific angle toward the visual marker at the edge of the swarm is indeed remarkable. However, the claim that mosquitoes then move toward a center line above the marker raises questions: how is this line perceived, and what explicit evidence in the data or stimulation suggests such a rule? Would it not be more parsimonious to assume that mosquitoes accelerate, after a saccade, toward areas of higher relative density and perform saccades only at the swarm edges?

   I think the comparison between data and simulation in Figure 5E also supports the notion that mosquitoes are likely to move towards higher densities/center, especially as the model does not fully capture movements in the most dorsal horizontal planes (highlighted in the blue box).

   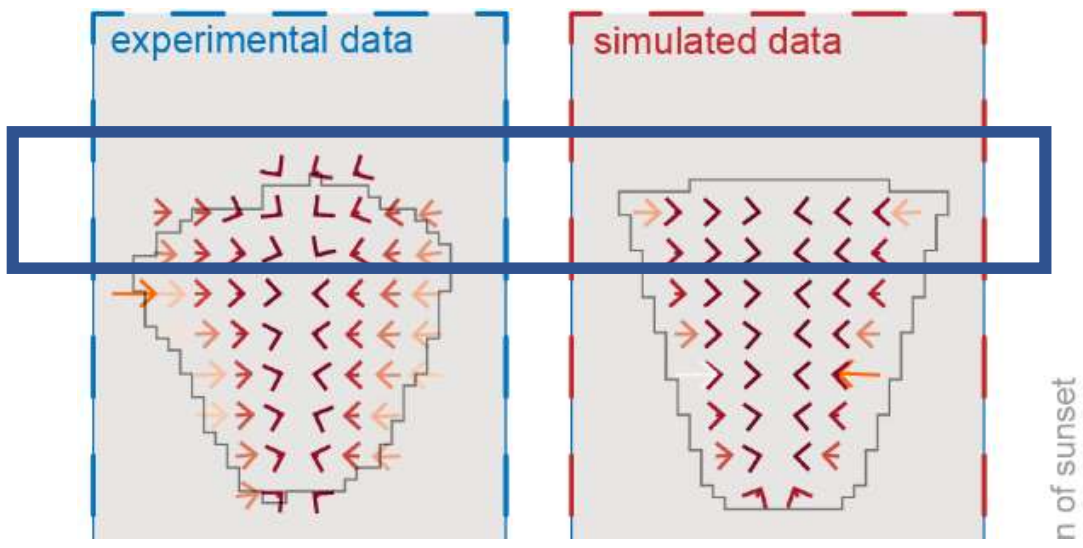

   Also, authors define this rule cue as non-social. Because the post-saccade travel directions were fitted from the real data collected in the presence of both environmental and social (at least visual) cues, it is unclear to me how social cues can be discounted or how causality can be attributed exclusively to environmental factors.

2. **Alignment** is defined in Figure 4 as “along the sunset axis” and in the Discussion as “they align their flight direction along a horizontal axis normal to the sunset horizon.” It would be helpful if the authors speculated on how mosquitoes compute such an axis. Many insects use the sun as a navigational cue, either by its direct position or by its polarization pattern. If the light source here was not polarized, mosquitoes must have been using the sun as a point source. This raises the question of how a point source could be used for alignment.

   It would also be helpful to test model performance in the absence of alignment.

   Animals can be very sensitive to subtle environmental biases. Despite the authors’ careful experimental design, a minor possibility of a ‘side bias’ remains, particularly since the paradigm was tested in a single configuration. Therefore, one has to be cautious, if the mosquito swarm is actually aligning towards the light source, but not to an overlooked external stimuli.

The authors report that mosquito swarming is scale invariant. Could they clarify how this finding aligns with the well-established observation that many boid or SPP models undergo density-dependent phase transitions, shifting from disorder to order as group size increases?

While the authors report scale invariance, it would be valuable to test this experimentally. I fully appreciate the challenges involved in collecting these data, which understandably justified focusing on smaller groups to reconstruct individual trajectories. That said, the current density range is severely limited. Additional experiments across a broader range of group sizes using swarm shape alone as the readout could provide important validation. If the authors' claims hold, the swarm shape should remain consistent until repulsive forces dominate. Where should repulsion dominate could be extractable from the simulations.

It would be beneficial to display individual trajectories from the simulations (at different horizontal planes) and compare them with the real data, as the modeling is at the microscopic level. Still, the reporting primarily focuses on swarm shape and representative group-level accelerations.

If the environment truly dominates and social cues play a negligible role, single mosquito trajectories should be indistinguishable from those recorded in a swarm. Since a single male mosquito initiates a swarm, meaning that such data is indeed available to the authors and they could compare individual trajectories over the visual marker to those of swarming groups.
