# Supplementary material for: The complex swarming dynamics of malaria mosquitoes emerge from simple minimally-interactive behavioral rules
Source: PLoS Comput Biol. 2026 Aug 26;22(8):e1014685. doi: 10.1371/journal.pcbi.1014685 (PMC13529247; doi:10.1371/journal.pcbi.1014685)
Supplement: Supplementary file 2 [file pcbi.1014685.s012.pdf]

Editor comments:

Comment 0.1: After careful consideration, we feel that it has merit but does not fully meet PLOS Computational Biology's publication criteria as it currently stands. Therefore, we invite you to submit a revised version of the manuscript that addresses the points raised during the review process.

Reply 0.1: We thank the editor for their interest in publishing our manuscript in PLOS Computational Biology. As requested, we have address the reviewers' comments and revised the manuscript accordingly.

Comment 0.2: Additional Editor Comments :

Three expert reviewers have carefully considered your manuscript and returned a range of useful comments and suggestions. Some of these are quite major and directly relate to the interpretation of the experimental data and so what inference we can make from your findings. Since most reviewers agreed the work was potentially of interest, I am willing to suggest "Major revisions", but the reviewers' comments must be dealt with through substantial revisions before the MS will be sent back out for review.

In particular, please ensure you:

Be clearer about the source of the data, and when figures are modified versions of elsewhere.

Address concerns about the comparison between leks and other types of collective motion, as it impacts on generality of findings.

Re-order to have Methods following Introduction, and be clear on sample sizes throughout.

Consider the other interpretations for your findings the reviewers suggest, and in particular deal with concerns of reviewer #2 that your results might be an artefact of your design.

Failure to robustly address these and other comments will lead to a desk rejection.

Reply 0.2: We thanks the editor for the detailed instructions and for giving us the chance to resubmit our revised manuscript. We made our best to answer the reviewers comments and revised our manuscripts to clarify and improve it.

In our revised manuscript, we now explicitly clarified the source of the data (from Poda et al. 2024), and clarified how this computational modelling study is distinct from the previous experimental paper.

We addressed the reviewer concerns about the comparison between leks and other type of collective motion, highlighting how mating swarms differs from fish schooling, bird flocking and the collective behavior of eusocial insects. We also now make explicitly clear how mating swarms are a widespread

and understudied collective behavior in insects, and that consequently our findings are likely relevant for diverse range of insect taxa.

As requested, we moved the material and method section, originally in the supplementary material, to the main text between the introduction and results sections. We also moved Fig. S8-9 to be Fig. S1-2 to insure that supplementary figures are referred to in sequential order. We now explicitly define the sample sizes, within the text and each figure. Several other minor edits were made to assure continuity and clarity.

We addressed the concerns of reviewer #2 regarding the size of the flight arena used in the swarming experiments. For this, we computed the distances between saccadic positions of mosquitoes and the flight arena boundaries (in new Fig. S11). Moreover, we added a paragraph in the results and in the discussion about how the laboratory environment may influence mosquito swarming behavior (lines 429-437 and 537-549). See also our detailed answer in reply 2.2.

Comment 0.3: Journal Requirements:

1) We ask that a manuscript source file is provided at Revision. Please upload your manuscript file as a .doc, .docx, .rtf or .tex. If you are providing a .tex file, please upload it under the item type u2018LaTeX Source Fileu2019 and leave your .pdf version as the item type u2018Manuscriptu2019.

2) Please provide an Author Summary. This should appear in your manuscript between the Abstract (if applicable) and the Introduction, and should be 150-200 words long. The aim should be to make your findings accessible to a wide audience that includes both scientists and non-scientists. Sample summaries can be found on our website under Submission Guidelines:

https://journals.plos.org/ploscompbiol/s/submission-guidelines#loc-parts-of-a-submission

3) Your manuscript is missing the following sections: Introduction, and Methods. Please ensure all required sections are present and in the correct order. Make sure section heading levels are clearly indicated in the manuscript text, and limit sub-sections to 3 heading levels. An outline of the required sections can be consulted in our submission guidelines here:

https://journals.plos.org/ploscompbiol/s/submission-guidelines#loc-parts-of-a-submission

4) Please upload all main figures as separate Figure files in .tif or .eps format. For more information about how to convert and format your figure files please see our guidelines:

https://journals.plos.org/ploscompbiol/s/figures

5) We have noticed that you have a list of Supporting Information legends for (Supplementary Videos, Data S1, Data S2, and Code S1) in your manuscript. However, there are no corresponding files uploaded to the submission. Please upload them as separate files with the item type 'Supporting Information'.

6) Please amend your detailed Financial Disclosure statement. This is published with the article. It must therefore be completed in full sentences and contain the exact wording you wish to be published.

7) State what role the funders took in the study. If the funders had no role in your study, please state: "The funders had no role in study design, data collection and analysis, decision to publish, or preparation of the manuscript."

8) Please ensure that the funders and grant numbers match between the Financial Disclosure field and the Funding Information tab in your submission form. Note that the funders must be provided in the same order in both places as well. Currently, "the Institut de Recherche our le Développement and the government of Burkina Faso" are missing from the Financial Disclosure field.

Note: If the reviewer comments include a recommendation to cite specific previously published works, please review and evaluate these publications to determine whether they are relevant and should be cited. There is no requirement to cite these works unless the editor has indicated otherwise.

Reply 0.3: Here is a point by point reply to the editor's comment regarding journal requirements:

1) We have uploaded a .doc manuscript source file for this revision. We highlighted the revised manuscript section in blue.

2) We have now included an Author summary in our manuscript and extended the abstract to better fit the journal requirements.

3) We have now included an "Introduction" and a "Material and Methods" sections in the main text. We also made sure to use consistent heading levels and style thorough the text.

4) We uploaded figures as .eps together with the manuscript.

5) Supporting datasets, Videos and codes can be found on our Dryad repository associated with this manuscripts: http://datadryad.org/stash/share/yyYPpUsKvRzBFxRI89pantIC9Fp19PP3YDpfPi9TZ6o. We clarified this in the "Data and materials availability" section.

6) We amended our detailed Financial Disclosure statement.

7) We included the following statement in our Funding section: "The funders had no role in study design, data collection and analysis, decision to publish, or preparation of the manuscript".

8) We ensured that the funders and grant numbers match between the Financial Disclosure field and the Funding Information tab in our submission form.

Review comments and replies

Reviewer: 1

Comment 1.1: Cribellier et al. use a state-of-the-art tracking system to study mosquito swarming behavior. The premise of the paper is that this behavior can be explained by simple rules, similar to those found in boid or zonal SPP models, which are commonly used to model collective motion. Crucially, the paper proposes a discrepancy: the authors argue that such simple rules are environmentally driven rather than resulting from interactions within the group. The manuscript begins with a description of the experimental paradigm and observed collective behavior (a concise version of the behavior-focused bioRxiv submission by Poda et al.), followed by model fitting and an exploration of the different models for this behavior.

Reply 1.1: We thank the reviewer for their accurate description of our study as well as for the constructive feedback and comments that helped us improving the manuscript. Also, we thank the reviewer for the time and effort put into reviewing our manuscript. See below out point-by-point reply to all comments and suggestions. We hope reviewer 1 will find our edits and revisions to be satisfactory.

Comment 1.2: Lekking and Comparisons to Other Collectives

Mosquito swarming is essentially a form of lekking, a mating behavior observed in many animals, in which males aggregate in a defined space. The advantage of leks is that such aggregates are stable in space and time, allowing females to join. Such stability can increase overall mating success through higher aggregate density, female mate choice, and male–male competition. In this regard, at least categorically, lek mating differs from the collective behaviors mentioned by the authors, such as fish schools and bird flocks, which represent the directed motion of groups that rely on conspecific interactions. Therefore, a direct comparison between two categories of collective behavior may risk overstating the similarities. In particular, the attempt to generalize mosquito lekking to insect collective motion more broadly, and to suggest that insect swarms are fundamentally distinct because they rely primarily on environmental cues, seems open to question. Other insect collectives, such as eusocial insects or locusts, exhibit definitely directed collective motion across considerable distances (thus less likely to be bounded by environmental clues). There, directionality indeed arises from social cues, such as pheromones; social rules govern the behavior, such as conspecific pursuit. Instead of lek mating, such insect swarms provide more suitable basis for comparison with the vertebrate collectives mentioned above.

I would therefore recommend substantial reconsideration regarding the extent to which mosquito swarming can be taken as case.

Reply 1.2: We thank the reviewer for their insightful comments, which we fully agree with. There are fundamental differences between the lekking of mosquitoes and most flocking of birds or schooling of fishes, as well as swarming of other insect species such as eusocial insects or locusts. We also agree that these nuances were not highlighted properly in our previous manuscript.

To correct this, we revised several sections of our manuscript, including the introduction and discussion section (lines 62-69 and 599-612, respectively). Here, we now introduce and discuss the concept of lekking swarms, and how these differ from other collective behaviors (bird flocking, fish schooling and other types of insect swarming). Additionally, we revised the section comparing mosquito swarming to other insect collective behaviors to better capture the diversity of these behaviors and emphasize that mosquito swarming most closely resembles lekking in other insects.

In that context, we believe that it is still valuable to compare mosquitoes swarming with other collective behaviors like flocking or schooling, to assess how and why the underlying flight behavior might differ. In fact, previous research has suggested that swarming mosquitoes follow behavior rules similar to those of birds or fishes [1], and thus this comparison is valuable for highlighting any similarities and differences. In our revised manuscript, we now better highlight the relevance and limitations of these comparative aspects of our study (lines 599-612); we hope that the reviewer agrees with this more balanced discussion.

Comment 1.3: Adaptation of Boid Models

By invoking fish schooling and bird flocking, the authors adapt boid models to mosquito swarming behavior. Boid (and related zonal) models are based on differential weighting (or zones) of avoidance, alignment, and attraction, in order of increasing distance from the focal individual. Classically, all three rules involve instantaneous adjustment to neighbors' behavior. In this manuscript, only repulsion is defined in that classical sense.

1. **Attraction** is described in Figure 4 as "toward the swarm center" and in the Discussion as "the swarming mosquitoes are attracted towards a vertical center line above the visual ground marker." The observation that mosquitoes turn at a specific angle toward the visual marker at the edge of the swarm is indeed remarkable. However, the claim that mosquitoes then move toward a center line above the marker raises questions: how is this line perceived, and what explicit evidence in the data or stimulation suggests such a rule? Would it not be more parsimonious to assume that mosquitoes accelerate, after a saccade, toward areas of higher relative density and perform saccades only at the swarm edges?

I think the comparison between data and simulation in Figure 5E also supports the notion that mosquitoes are likely to move towards higher densities/center, especially as the model does not fully capture movements in the most dorsal horizontal planes (highlighted in the blue box).

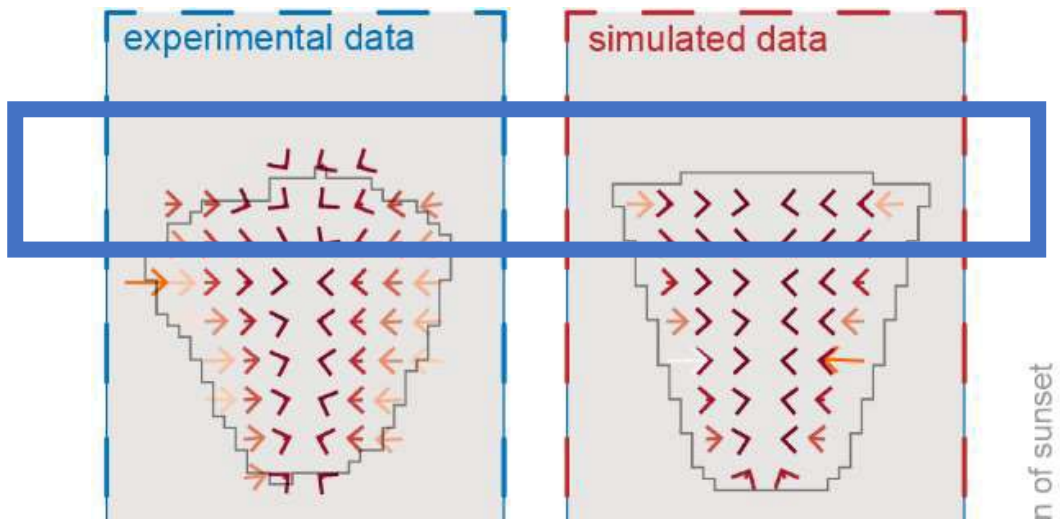

Also, authors define this rule cue as non-social. Because the post-saccade travel directions were fitted from the real data collected in the presence of both environmental and social (at least visual) cues, it is unclear to me how social cues can be discounted or how causality can be attributed exclusively to environmental factors.

Reply 1.3: We agree that our phrasing in the manuscript may have given the impression that mosquitoes perceive a "center line" directly, but this is not what we intended to imply. Our data and modeling instead indicate that mosquitoes fly toward this apparent vertical axis as an emergent outcome of using environmental cues to orient their flight. Mosquitoes detect swarm boundaries by monitoring the viewing angle of the ground marker, and then perform a saccadic turn back toward the ground marker center. After turning, the agent randomly choose new azimuth and climb angles drawn from experimental distributions, producing flight paths biased toward the space above the marker and along the sunset axis. Thus, the apparent attraction to a "center line" arises indirectly from visual orientation to these environmental cues rather than perception of the line itself. We have clarified this thorough the manuscript.

We believe that it remains most parsimonious to assume that mosquitoes rely solely on environmental information. Maintaining position above the marker inherently requires continuous visual feedback on its position, thus why not using these cues also to inform the rest of swarm behavior? Estimating the swarm center from the positions of other males would still require knowledge of the swarm's location to avoid drifting away from it, while also being unreliable under low-light conditions and sparse densities given mosquitoes' limited visual acuity. Moreover, swarming kinematics were consistent across swarm sizes from 1 to 26 males (Fig. S8, and Videos S1-S3), and even solitary males exhibited similar flight behavior—strongly suggesting that social cues played a negligible role in our mosquito swarms.

Regarding the reviewer's note on Fig. 5E, we acknowledge that the model slightly underrepresents motion near the upper swarm boundary. As discussed in our reply 2.6, this results from our simplified binary treatment of the swarm boundary ("in" or "out"), whereas real boundaries are less clearly defined. Nonetheless, the model reproduces the main observed flight features without requiring social interactions.

Comment 1.4: 2. **Alignment** is defined in Figure 4 as "along the sunset axis" and in the Discussion as "they align their flight direction along a horizontal axis normal to the sunset horizon." It would be helpful if the authors speculated on how mosquitoes compute such an axis. Many insects use the sun as a navigational cue, either by its direct position or by its polarization pattern. If the light source here was not polarized, mosquitoes must have been using the sun as a point source. This raises the question of how a point source could be used for alignment.

It would also be helpful to test model performance in the absence of alignment.

Animals can be very sensitive to subtle environmental biases. Despite the authors' careful experimental design, a minor possibility of a 'side bias' remains, particularly since the paradigm was tested in a single configuration. Therefore, one has to be cautious, if the mosquito swarm is actually aligning towards the light source, but not to an overlooked external stimuli.

Reply 1.4: We agree with the reviewer that, in absence of specific experimental tests to prove that mosquitoes aligned their flight trajectories using the simulated sunset point source, we cannot entirely rule out that mosquitoes used other external stimuli for such alignment. We have edited the text to make this clearer, whereby we now mention that other factors might have induced this alignment in flight behaviors (lines 496-506). Because it is not yet exactly clear which cues mosquitoes are using to align their flight path, we prefer to not speculate about that in the text. But we believe that if mosquitoes use the simulated sunset point source as an alignment cues, they probably do so by keeping the swarm marker below them (so using it to know if they are in the swarm), and preferentially flying towards or away from the sunset point source. A way to test this hypothesis could be to move the sunset point source and see if flight directions keep aligning with it. In our revised manuscript, we now suggest such approach for assessing these aspects in future studies (lines 501-502).

Comment 1.5: The authors report that mosquito swarming is scale invariant. Could they clarify how this finding aligns with the well-established observation that many boid or SPP models undergo density dependent phase transitions, shifting from disorder to order as group size increases?

While the authors report scale invariance, it would be valuable to test this experimentally. I fully appreciate the challenges involved in collecting these data, which understandably justified focusing on smaller groups to reconstruct individual trajectories. That said, the current density range is severely limited. Additional experiments across a broader range of group sizes using swarm shape alone as the readout could provide important validation. If the authors' claims hold, the swarm shape should remain consistent until repulsive forces dominate. Where should repulsion dominate could be extractable from the simulations.

Reply 1.5: We thank the reviewer for its useful suggestion to perform experiments and analyses specifically focused on the effect of density on swarming behavior. In our study, we recorded mosquito

flight behavior from the onset of swarming (single male mosquito) up until fully formed swarms of up to 26 simultaneously swarming individuals. So, our experiments already provided us with the variations in swarm density needed for the analysis suggested by the reviewer.

When doing so, we found that swarming behavior did not vary with swarm sizes from one to 26 males; Fig. S8). In classical self-propelled particle (SPP) or BOIDS frameworks (e.g., the Vicsek model), density-dependent phase transitions from disorder to ordered motion occur primarily via alignment interactions among neighbors [2]. Because in our case alignment and collective directionality among males appear negligible, the conditions for such transitions are not met in our system.

Note that swarm densities in our experiments remained very low with respect to densities observed in bird flocks or fish schools. In fact, in our largest swarms of 26 individuals, the nearest-neighbor distance was on average 20.7 body-lengths, whereas in bird flocks or fish schools these are respectively in the range of ~3-7 or ~1-2 body lengths [3,4]. This could suggest that our swarms were too small for triggering the transition from disorganized to organized swarms, but this is not supported by literature. In fact, previous studies found that larger field swarms consist not only of more individuals, but these swarms were most of the time of larger volumes [5–7]. As a result, swarm density (number of individuals per unit volume) remains similarly low. Under such low-density, minimal-interaction conditions, the behavioral rules appear to remain constant, which may explain why we observe invariance in swarm shape and kinematics across the tested group sizes.

As suggested by the reviewer, we now explicitly discuss our revised analysis on the effect of swarm size on swarm kinematics, showing that these are largely independent on swarm size. We now also discuss the cause of this independence, which is due to the relatively low densities in mosquito swarms (lines 429-437 and 537-549).

Comment 1.6: It would be beneficial to display individual trajectories from the simulations (at different horizontal planes) and compare them with the real data, as the modeling is at the microscopic level. Still, the reporting primarily focuses on swarm shape and representative group-level accelerations. If the environment truly dominates and social cues play a negligible role, single mosquito trajectories should be indistinguishable from those recorded in a swarm. Since a single male mosquito initiates a swarm, meaning that such data is indeed available to the authors and they could compare individual trajectories over the visual marker to those of swarming groups.

Reply 1.6: Our modelling framework was intentionally designed to capture the minimal set of mechanisms necessary to reproduce the emergence and structure of mosquito swarms, rather than to generate realistic-looking individual trajectories. To this end, we adopted a simple rule set - straight flight segments and sharp turns triggered by the visual marker - that captures the essential kinematic features underlying swarming while avoiding unnecessary parameterization. Adding noise or curvature

to reproduce more natural-looking flight paths would increase model complexity without improving explanatory power, as our goal was to test whether environmental visual cues alone can account for the observed group-level swarm organization.

Consequently, individual simulated trajectories are intentionally simpler than those measured experimentally. We agree, with the reviewer's reasoning that if environmental structure dominates and social cues play a negligible role, single-mosquito trajectories should resemble those observed in swarms. To test this key implication experimentally, we directly compared swarming characteristics across a wide range of swarm sizes (1–26 males; Fig. S8 and Videos S1-S3). These analyses revealed no significant differences in swarm geometry or kinematic statistics with group size, suggesting that the same behavioral rules govern both solitary and group swarming. We now emphasize this result more explicitly in the revised manuscript (lines 429-437 and 537-549). Finally, individual trajectories of real and simulated mosquitoes can be compared in the supplementary videos S3 and S4.

Reviewer: 2

Comment 2.1: I am afraid the manuscript is not ready to be published in Plos Computational Biology. I do not recommend the publication.

My concerns are mostly on the interpretation of the experimental data, which I believe are overinterpreted, and on the conclusions the authors draw from the comparison with the boids model.

Reply 2.1: We thank the reviewer for the time and effort put into reviewing it. Below, we discuss the reviewer's concerns and suggestions to further improve our manuscript. See below out point-by-point reply to all comments and suggestions.

Comment 2.2: DATA INTERPRETATION

Two main features of mosquito behavior are considered crucial in the manuscript. The first is the preferred direction of flight, orthogonal to the sunset horizon. The second is that mosquitoes perform saccades at the border of the swarm, due to a visual interaction (defined by a specific visual range) with the marker.

My concern is that both features may be artifacts of the experimental setup. The direction orthogonal to the sunset horizon corresponds to the shortest side of the cage. Therefore, an alternative interpretation of the data, different from that of the authors, is that mosquitoes are not completely free to move in that direction and that the cage is affecting the behavior. For instance mosquitoes may be induced to perform fast 180deg turns (saccades), to avoid the wall and redirect towards the central, open area of the cage. With a similar reasoning, mosquitoes at the borders of the swarm may be induced to perform saccades, not because of a visual interaction with the marker, but because of a physical constraint of the cage itself.

To determine which of the two alternatives (the one proposed by the authors and the one described above) is the more realistic, the direction orthogonal to the sunset horizon must be separated from the shortest side of the cage, and the swarm boundaries must be separated from the cage boundaries. These variables cannot be decoupled in the data presented here.

Therefore, authors need to perform additional experiments: 1- with the sunset in a different position/direction to test whether the preferred direction is related to the sunset; 2- with different markers (shape and size) to validate the proposed visual interaction.

Reply 2.2: We thank the reviewer for raising this important point. We agree that the observed flight direction and saccadic maneuvers could, in principle, be influenced by the physical constraints of the flight arena. To address this, we performed a dedicated analysis quantifying the proximity of mosquitoes to the arena walls during saccadic turns (see new Fig. S11).

Our results show that mosquitoes turned at average distances of 45 ± 8.5 cm from the side walls and 23 ± 7.1 cm from the front/back walls—equivalent to 75 and 38 body lengths, respectively. These distances are an order of magnitude greater than the inter-individual distances that trigger collision avoidance (~2.5 body lengths; Fig. 5K, [8]).

Additionally, the preferred flight direction—orthogonal to the sunset horizon—aligns with the short axis of the arena (y-axis), which is counterintuitive if wall effects were dominant. If cage geometry were driving the behavior, we would expect alignment along the long axis (x-axis), which is not observed.

Together, these findings strongly suggest that wall avoidance does not explain the observed saccadic maneuvers or flight orientation. Instead, the data support our interpretation that these behaviors are visually mediated interactions with the marker.

We acknowledge the reviewer's suggestion for further experiments to disentangle these effects. While such tests (e.g., varying sunset direction or marker shape) would be valuable for refining our understanding of swarm geometry, they fall outside the main scope of this study. Our primary aim is to identify the behavioral rules underlying mosquito swarming, rather than to characterize the exact shape or orientation of the swarm. Moreover, our newly-added analysis based on current data already provide strong evidence that the observed behaviors are not artifacts of the experimental setup. We have added this new analysis and expanded the discussion accordingly (lines 420-426 and 564-570; Fig. S11).

Comment 2.3: BOIDS MODELS

To the best of my knowledge, the dominant term in boids models is the alignment interaction, as in other interacting models, like Vicsek's. Repulsion and attraction are essentially needed to have realistic simulations and applications in robotics.

The low relevance of repulsion is indeed in line with what the authors find in their data, where repulsion events are rare. In regard to the attraction term, the situation is more subtle. Boids model considers attraction towards the average position of the neighbors. In large systems, this means that particles in the bulk will not feel the effect of the attraction. The attraction is instead dominant for particles at the periphery, and attraction is actually needed to avoid a loss of cohesion.

In the simulation presented in the manuscript, all the particles are essentially at the periphery of the swarm, since there are only 10 particles in the group. As a result, they are all influenced by the attraction toward the average position of the other ones, which due to the limited setup, always coincides with the position of the landmark. It is then not possible to discriminate between the presence of an individual attraction toward the marker, as suggested by the authors, or an attraction toward the other members of the group. This ambiguity should be discussed within the manuscript.

Reply 2.3: We appreciate the reviewer's insightful comment. Indeed, distinguishing between attraction to conspecifics and attraction to an environmental marker is challenging in collective behavior studies.

Two key findings support that the marker is the primary swarm attractor:

1. Stable swarm position: Swarms consistently remained above the marker for extended periods. If attraction were primarily to conspecifics, we would expect positional drift over time—especially given that our chamber design allows for such movement. This drift was never observed.
2. Swarming by single individuals: We recorded flight kinematics from the onset of swarming (single mosquito) to fully developed swarms (up to 26 individuals; Fig. S8). Even solitary males exhibited sustained swarming above the marker, with kinematics comparable to those in larger swarms (see Reply 1.5 for details). This suggests that attraction to the marker is independent of swarm size.

Based on these results, attraction to the marker remains the most parsimonious explanation for swarm cohesion. To test this, we developed an agent-based model using the marker as the sole attractor, with collision avoidance as the only conspecific interaction. This minimal model successfully reproduced swarm kinematics across group sizes, indicating that explicit attraction between individuals is not required.

While we cannot entirely exclude conspecific attraction, our data show it is not necessary to explain mosquito swarming. We have updated the manuscript to include these analyses and expanded the discussion on the model's assumptions and limitations (lines 429-437 and 537-549).

MINOR COMMENTS

Comment 2.4: 1- The number of mosquitoes in the analyzed swarms should be clearly stated. At the beginning of the Experimental section, authors mention that they reconstructed 938 tracks. In SI, section "Experimental conditions", they describe their experimental procedure and refer to the number of mosquitoes released in the cage (30 or 50 depending on the experiment). It is only in the description of the simulations, SI Section "Agent-based model simulations", that they finally state that the data in the manuscript refer to swarms of approximately ten mosquitoes. It would be better to clarify from the beginning of the manuscript that the data are 938 reconstructed tracks, corresponding to 18 swarms of approximately ten mosquitoes.

Reply 2.4: As requested by the reviewer, we now clarify in detail the number of swarming experiments performed (six swarms with three recordings per swarm), and the number of swarming mosquitoes per swarm (from 1 to 26 individuals) thorough the manuscript. Moreover, we now explicitly state the sample sizes used for each figure in its figure legend. We also discussed how the number of swarming mosquitoes did not seem to affect their swarming behavior (lines 537-549).

Comment 2.5: 2- The discussion section appears too general and superficial. For instance, the last sentence on vector control should be removed or expanded. In principle any study on mosquitoes may be considered useful for vector control. The authors need to be specific on how their findings could be useful for applications in vector control. Another example in this direction is the paragraph at line 10 pag.7 (second last paragraph) of the discussion section, about male-female and male-male interaction. This paragraph does not add anything to the overall message of this work, and should be removed or revised.

Reply 2.5: As requested by the reviewer, we extended the discussion section in the revised manuscript. Hereby, we including a comparison of the studied mosquito swarming with other group behaviors (lines 599-612), we revising the paragraph discussing how our study informs us on male-female interactions in mosquito mating swarms (lines 590-598), and we expended the part of the discussion about vector control (lines 613-625). We hope that the reviewer is satisfied with the extended discussion section.

Comment 2.6: 3- Authors do not address potential effects of the laboratory environment on mosquito behavior.

Reply 2.6: As mentioned in our Reply 2.2, we have added a dedicated analysis addressing potential effects of the laboratory environments on the recorded mosquito behaviors. We discuss this in the methods, results and discussion sections of the revised manuscript (lines 420-426 and 561-570; see also Fig. S11).

Comment 2.5: 4- Rules defining the models should be at least mentioned in the main text. Otherwise the reader is forced to go to SI.

Reply 2.5: We have now included the material and methods section in the main text, thus resolving this issue.

Comment 2.6: 5- The comparison between model and data shows that simulations produce saccades symmetric on the plane xy and yz, against a clear asymmetry in the data. The authors should comment on this discrepancy. The same symmetry/asymmetry is present in the comparison of the straight flight.

Reply 2.6: We believe that the reviewer refers to results presented in Fig. S4. In that figure, we compare the 2D heat maps of mean angular speed or of saccade-to-straight flight ratio in the XY and YZ plans. When comparing the heatmaps from the experimental data and from simulated data, we see that both real and simulated mosquitoes have on average higher angular speeds on the boundaries of the swarm, thus showing high proportion of saccadic maneuvers at the edges, and high proportion of straight flights in the middle of the swarm. These 2D distributions of both experimental and simulated data follow a

clear axis symmetry around the central vertical axis of the swarm (Fig. S4). Therefore, it is unclear to us which asymmetry the reviewer refers to.

There are, however, key differences between the distributions of the experimental data and simulated data. Most importantly, in the simulations we observe high angular speed and high proportion of saccades around the swarm boundaries (Fig. S4c,d and Fi. S4g,h). This is due to a simplification of our agent-based model where mosquitoes will only trigger a saccade if they are going to exactly cross the swarm boundaries (i.e. binary threshold), while in reality they can trigger a saccade a bit before or after crossing the boundary. This results for the experimental swarms in more diffused distributions of saccades in space (Fig. S4c,d and Fi. S4g,h) and less defined swarm boundaries (as visible on Fig. 5C,D).

We have now included a comparison of the experimental and simulation results in the discussion (lines 520-536 and 571-578), highlighting these similarities and differences.

Reviewer: 3

Comment 3.1: This work describes the swarming of malaria mosquitoes based on individual 3D tracks in the lab and investigates the underlying behaviour rules of their swarms using an agent-based model. I find the paper clear and concise, and a very nice fit to the journal. It is well-written and with nice visualization of results. I have however a few remarks and questions that I think should be addressed before the paper's publication, and some comments that I think can increase the paper's contribution and impact. Please find them below:

Reply 3.1: We thank the reviewer for the accurate summary of our study, for their kind words, and for the time and efforts put into reviewing our paper. We answered the reviewer's questions and suggestions below.

Comment 3.2: Why there are no methods in the main text? Some experimental conditions should be mentioned in the results text, especially how many mosquitoes were tracked and for what duration. Same for some main aspects of the simulations.

Reply 3.2: As requested by the reviewer, we moved the material and methods in the main text between the introduction and results section. Additionally, we clarified now how many mosquitoes were present in the swarm (lines 352-355), and discuss how swarming behavior was found to be independent of the number of swarming mosquitoes (lines 429-437 and 537-549).

Comment 3.3: I understand this paper is an extension of the Poda et al. 2024 work. Given the overlap, I think that the authors should more clearly state (perhaps even in the introduction/results) and discuss in the end the differences/extensions in empirical (especially for sections 'Flight kinematics of individual swarming mosquitoes' and 'Variations in flight kinematics within the swarm volume') and modelling findings (since a different model was used in the previous study).

Reply 3.3: As suggested by the reviewer, we now explicitly mention in the introduction that this paper was extending the data analysis of Poda et al. 2024 (line 92-93), and we clarified throughout the text how our current modelling work differs from and complements to that of the previous study. Finally, by including the Material and Methods in the main text, we now also explicitly discuss how the model developed in the previous paper has been used as input for our agent-based model (lines 260-261). We thank the reviewer for this suggestion, because adding these aspects significantly improve the readability of the manuscript.

Comment 3.4: Fig. 1A is exactly the same as in ref [37], Poda et al. 2024, without a reference in the caption. Not sure what's the journal's policy is on this.

Reply 3.4: We added a reference to Poda et al. 2024 in the figure caption to clarify this.

Comment 3.5: The comparison with other species could be made a bit more specific, e.g., pg.7 line 5, what different environmental cues? Are there any relevant findings from other swarming insects?

Reply 3.5: We have now included a new paragraph in the discussion section to compare the swarming of mosquitoes with collective behaviors of other animals (lines 599-612).

Comment 3.6: I find the number 4.5% reduction of the viewing angle as the trigger to a turning manoeuvre a bit too specific, perhaps the authors could elaborate more in their Discussion about it.

Reply 3.6: We agree with the reviewer that the 4.5% threshold is quite specific and consequently a bit arbitrary. This number was found to describe well the 3D shape of the swarm encompassing 95% of mosquito swarming positions in our previous study (Poda et al. 2024). In reality, the boundaries of the swarm are not so clearly defined (as can be seen on Fig. 5A,B), and mosquitoes are found to perform their saccadic maneuver at a range of viewing angle deviations (Fig. S11*d*).

For simplicity, we used a single threshold of 4.5% in our model, which yields a high similarity between the simulated and experimental swarms (Fig. 5 and S3-S7). That said, we do not believe that the general swarming kinematics is sensitive to the exact threshold, and similar swarms would be produced at other thresholds (albeit slightly smaller or larger in size). We included new sentences in the discussion section to address this topic and discuss differences between experimental and simulated results (lines 520-536).

Comment 3.7: Model description: As I mentioned already above, some more details about the simulations should be added in the main text. A table with all the parameters and their default values should also be added in the Supplementary. Additionally, despite the model description being clear, I would recommend that the authors could at least use more terminology from the ODD protocols (to not write a full one) [1]. This will support both the reproducibility of the agent-based model as well as strengthen its link to the literature.

References:

[1] Grimm, Volker, Steven F. Railsback, Christian E. Vincenot, Uta Berger, Cara Gallagher, Donald L. DeAngelis, Bruce Edmonds et al. "The ODD protocol for describing agent-based and other simulation models: A second update to improve clarity, replication, and structural realism." Journal of Artificial Societies and Social Simulation 23, no. 2 (2020).

Reply 3.7: We have now included the complete material and methods section in the main text. Moreover, we added a new table to the supplement, in which we explicitly define all parameters and their values used in our agent-based model (Table S1). For this, we used the terminology of the ODD protocol in the main text (lines 261-312) and in the table S1, as recommended by the reviewer. We refer to this table S1 on line 296 of the revised manuscript.

Comment 3.8: Pg 7 line 19: 'mosquito swarms as an ideal system for studying animal collective behavior' I found this opposing the conclusion that mosquito swarms are 'inherently different from flocking and schooling'. Perhaps the authors meant collective behaviour of insect swarms? Saying that, a paragraph on comparisons with findings in other insect swarms would be nice addition to the Discussion.

Reply 3.8: We clarified this paragraph and added sentences discussing the difference between mosquito mating swarms with other types of collective behaviors, including swarms of eusocial insects, bird flocks and fish schools. Moreover, we now discuss in a more balanced way how our research contributes to better understanding collective behaviors in general, and specifically mating swarming in insects (lines 599-612).

Comment 3.9: Maximum swarm size from Fig S7 seems to be 26, is this the maximum over the experiments or just the one used for the plot? In general, are the swarm sizes here reflecting the ones found in the field? Perhaps the authors could expand a bit on their discussion pg 6 line 44.

Reply 3.9: Yes, 26 individuals was the maximum number of mosquitoes that were swarming simultaneously (now Fig. S8). Swarm size in the field can vary greatly, from tens to several hundreds of mosquitoes [9–12]. As requested by the reviewer, we extended our discussion about swarm size (lines 429-437 and 537-549).

Comment 3.10: Fig. S4 f and h: there seem to be larger differences between empirical data and simulations here, perhaps the authors can provide some clarification of why this is the case in the caption or text.

Reply 3.10: It appears indeed that there is larger differences between empirical data and simulations on these top views of the climb angle between successive saccades (now Fig. S4f and h). This is for most part due to the relative simplicity of our model and simulations, whereby all simulated mosquitoes in the top or bottom of the swarm will turn directly down or up when they reach the swarm boundary, respectively. In reality though, this swarm boundary is not so strongly defined, and real mosquitoes exhibit a larger range of upward and downward directed turns. We could increase the fidelity of this

aspect of the model by adding more noise to the turn dynamics, but it would not change the general results. We discuss this limitation of our model and its effect on the results explicitly in the discussion section of our revised manuscript (lines 520-536).

Comment 3.11: Pg. 6, line 39: 'previously suggested apparent long distance interactions' I think that this statement needs references.

Reply 3.11: We added the required references [13–16].

Comment 3.12: Minor comments:

- pg. 3 line 40: unnecessary second 'and' ?
- pg. 4 line 19: 'remain'
- Ref 37: bioRxiv references instead of the published version
- title: 'emerge' rather than 'emerges' if it refers to the dynamics and not the swarming.

Reply 3.12: We corrected these mistakes. Again, we thank the reviewer for the thorough and constructive feedback.

References

1. Charlwood JD. Swarming and mate selection in Anopheles gambiae mosquitoes (Diptera: Culicidae). J Med Entomol. 2023;60: 857–864. doi:10.1093/JME/TJAD064

2. Lobaskin V, Romenskyy M. Collective dynamics in systems of active Brownian particles with dissipative interactions. Phys Rev E Stat Nonlin Soft Matter Phys. 2013;87. doi:10.1103/PHYSREVE.87.052135

3. Harpaz R, Tkačik G, Schneidman E. Discrete modes of social information processing predict individual behavior of fish in a group. Proc Natl Acad Sci U S A. 2017;114: 10149–10154. doi:10.1073/PNAS.1703817114/SUPPL_FILE/PNAS.1703817114.SM03.MP4

4. Hemelrijk CK, van Zuidam L, Hildenbrandt H. What underlies waves of agitation in starling flocks. Behav Ecol Sociobiol. 2015;69: 755–764. doi:10.1007/S00265-015-1891-3

5. Manoukis NC, Diabate A, Abdoulaye A, Diallo M, Dao A, Yaro AS, et al. Structure and Dynamics of Male Swarms of Anopheles gambiae. J Med Entomol. 2009. Available: https://academic.oup.com/jme/article/46/2/227/876108

6. Manoukis NC, Butail S, Diallo M, Ribeiro JMC, Paley DA. Stereoscopic video analysis of Anopheles gambiae behavior in the field: Challenges and opportunities. Acta Trop. 2014;132: S80-5. doi:10.1016/j.actatropica.2013.06.021

7. Poda SB, Nignan C, Gnankiné O, Dabiré RK, Diabaté A, Roux O. Sex aggregation and species segregation cues in swarming mosquitoes: Role of ground visual markers. Parasit Vectors. 2019;12: 589-. doi:10.1186/S13071-019-3845-5/TABLES/3

8. Gupta S, Cribellier A, Poda SB, Roux O, Muijres FT, Correspondence JAR. Mosquitoes integrate visual and acoustic cues to mediate conspecific interactions in swarms. Current Biology. 2024;0. doi:10.1016/J.CUB.2024.07.043

9. Diabate A, Baldet T, Brengues C, Kengne P, Dabire KR, Simard F, et al. Natural swarming behaviour of the molecular M form of Anopheles gambiae. Trans R Soc Trop Med Hyg. 2003;97: 713–716. doi:10.1016/S0035-9203(03)80110-4

10. Diabaté A, Yaro AS, Dao A, Diallo M, Huestis DL, Lehmann T. Spatial distribution and male mating success of Anopheles gambiae swarms. BMC Evol Biol. 2011;11: 184-. doi:10.1186/1471-2148-11-184/FIGURES/7

11. Birungi K, Mabuka DP, Balyesima V, Tripet F, Kayondo JK. Attributes of Anopheles gambiae swarms in South Central Uganda. Parasit Vectors. 2024;17: 149-. doi:10.1186/S13071-024-06132-9/FIGURES/10

12. Somda NSB, Poda BS, Sawadogo PS, Gnankine O, Maiga H, Fournet F, et al. Ecology of reproduction of Anopheles arabiensis in an urban area of Bobo-Dioulasso, Burkina Faso (West Africa): Monthly swarming and mating frequency and their relation to environmental factors. PLoS One. 2018;13: e0205966. doi:10.1371/JOURNAL.PONE.0205966

13. Cavagna A, Giardina I, Gucciardino MA, Iacomelli G, Lombardi M, Melillo S, et al. Characterization of lab-based swarms of Anopheles gambiae mosquitoes using 3D-video tracking. Sci Rep. 2023;13. doi:10.1038/s41598-023-34842-0

14. Kelley DH, Ouellette NT. Emergent dynamics of laboratory insect swarms. Sci Rep. 2013;3. doi:10.1038/srep01073

15. Shishika D, Manoukis NC, Butail S, Paley DA. Male motion coordination in anopheline mating swarms. Sci Rep. 2014;4: 1–7. doi:10.1038/srep06318

16. Attanasi A, Cavagna A, Del Castello L, Giardina I, Melillo S, Parisi L, et al. Collective Behaviour without Collective Order in Wild Swarms of Midges. PLoS Comput Biol. 2014;10. doi:10.1371/journal.pcbi.1003697
