# Supplementary material for: The complex swarming dynamics of malaria mosquitoes emerge from simple minimally-interactive behavioral rules
Source: PLoS Comput Biol. 2026 Aug 26;22(8):e1014685. doi: 10.1371/journal.pcbi.1014685 (PMC13529247; doi:10.1371/journal.pcbi.1014685)
Supplement: Supplementary file 3 [file pcbi.1014685.s013.pdf]

Editor:

- Artifacts caused by the experimental setup (e.g., cage geometry) cannot be ruled out based on the presented data. Proper controls for this would be essential. Ultimately, the conclusions based on the current data are too strong.

Authors:

To address the first point, we extended our computational and modelling analysis beyond the (Poda et al., 2024) dataset by incorporating a second, independent swarming-flight dataset (Feugère et al., 2022), and rewrote a significant part of the manuscript. This allowed us to evaluate the robustness of our findings across distinct laboratory environments, arena geometries, and marker configurations.

Comments:

The addition of **Feugère et al. (2022)** substantially reduces the concern that the observed swarm shape and organization are artifacts of a single arena geometry. Given the clear differences between the two setups, I think this new comparison removes most of the doubt that the main swarm features arise solely from the specific chamber used in Poda et al. In that sense, the added dataset is a meaningful and welcome strengthening of the manuscript

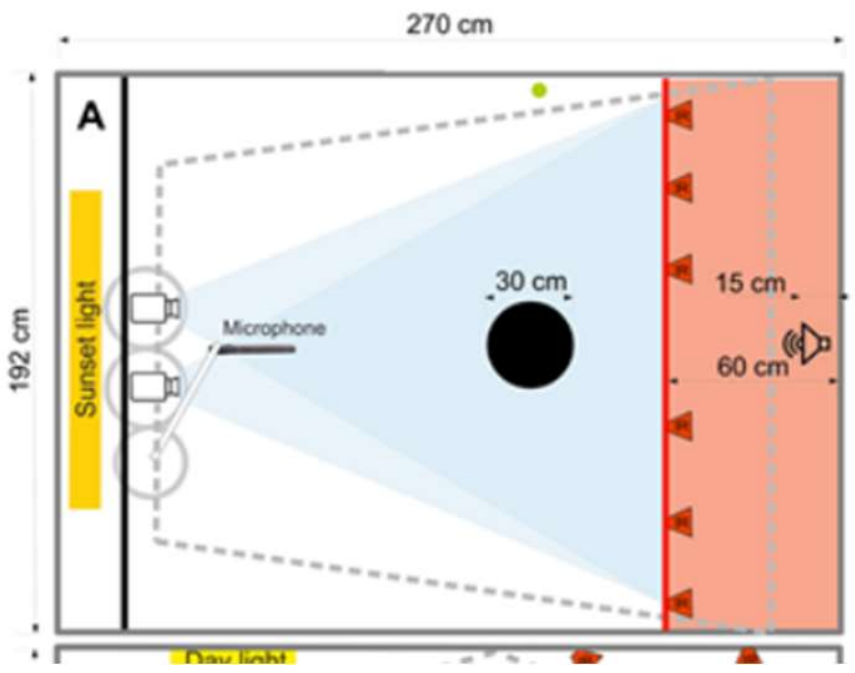

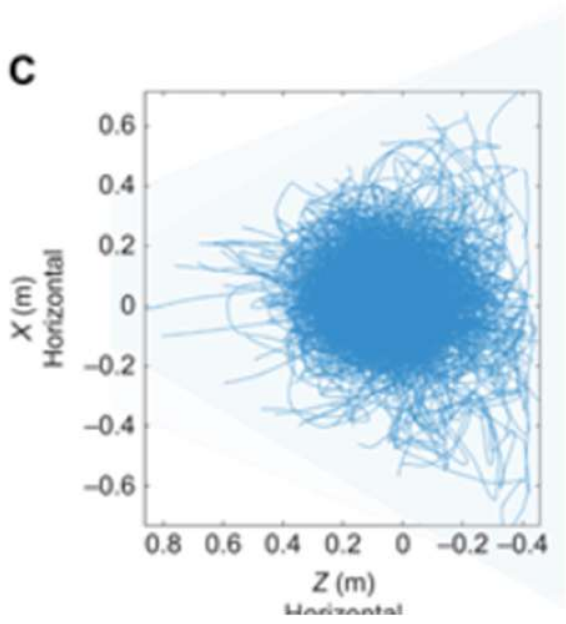

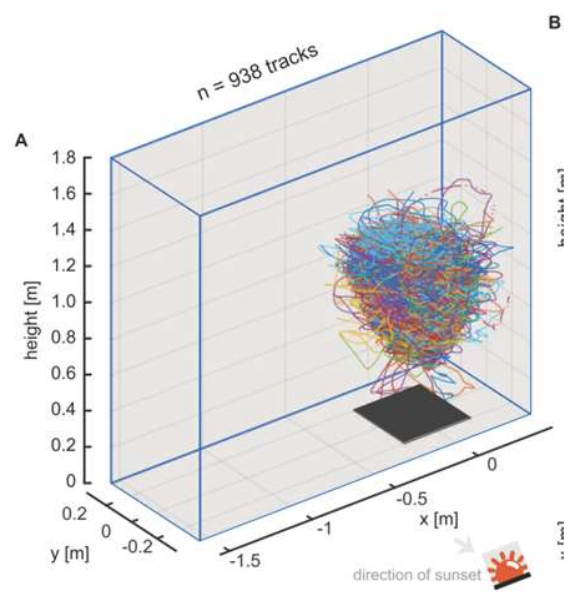

Nevertheless, revisiting the experimental design, I still find that one crucial aspect is missing. The very strong contrast of the dark swarm marker in a confined laboratory setting may not be fully ecologically representative. In this setup, there is effectively a single salient dark landmark available. In the field, by contrast, males may encounter multiple candidate landmarks, and under such conditions social recruitment or other collective effects could still play a role in determining where and how swarms form. Thus, while the present revision is clearly stronger, I think the authors have demonstrated reproducibility of similar mosquito

behavior in **two different laboratory contexts**, rather than having fully excluded experimental-context effects more generally.

Editor:

- The dynamic range of swarm sizes in the experiments is insufficient to support the current conclusions. Resolving these issues will require either additional experimental evidence or drastic changes to the main conclusions (see the specific suggestions of reviewer #2). The latter option would require changes that go beyond what can be done in a major revision and would fundamentally alter the manuscript.

Authors:

"Finally, we added a dedicated limitations section to the revised discussion, in which we explicitly describe the constraints of our study, most notably, the differences between laboratory and natural swarms, thereby directly addressing the core concerns raised by the reviewers."

(In text)

In the Poda dataset, swarm sizes ranged from a single initiator male (Video S1) to groups of up to 26 simultaneously swarming individuals (Videos S2–S3). This range corresponds to the modal 475 sizes reported for natural Anopheles mating swarms in the field, which commonly consist of several to a few dozen males [48–50], although much larger swarms, occasionally reaching hundreds to thousands of individuals, have been documented in some locations.

Comments:

While the authors did not perform additional experiments expanding the swarm-size range, I find their revised argumentation acceptable. They now acknowledge much more clearly the ecological range they are probing, and I think that is the correct way to handle this limitation.

More broadly, I think studying low- to mid-density swarms is valuable in its own right, not only because these may correspond to common field swarm sizes, but also because such events can represent the initial stage of larger and more persistent swarm formation. It is also entirely plausible that the effective rules differ in much larger swarms, where physical crowding, density-dependent effects, or other interactions could become more important.

Revisiting the paper, however I still find one point missing. The authors document in **Fig. S8** that, within the **Poda dataset**, the number of swarming mosquitoes ranged from **1 to 26**, and that across this sampled range they detected no clear effect of group size on several **individual flight-kinematics metrics**, namely **saccadic turn angle**, **straight-flight distance between successive saccades**, and **straight-flight duration**. This is useful and

should be stated clearly as a bounded result: within the sampled low- to mid-density range, the measured individual kinematics appear largely insensitive to swarm size. However, the manuscript does not provide an analogous quantification for **swarming duration**, i.e. neither a direct distribution of **full swarm lifetime** nor of **individual residence time** within the swarm. In other words, even if turn angle or straight-flight statistics are unchanged, swarm size could still matter at the level of how long individuals keep swarming and how long the swarm itself remains coherent. Quantifying these persistence-related measures would therefore provide a more complete test of whether swarm size truly has limited influence within the studied range.

In general, I also appreciate that the authors are sincere in acknowledging the limitations of the study. However, this caution is still expressed mainly in the **Discussion**, whereas the more prominent parts of the manuscript remain somewhat stronger in tone. For example, I think the **Abstract** could state the swarm-size scope even more explicitly, for instance emphasizing that they studied low-mid densities.

A further minor but important point concerns the repeated use of the term **"mechanistic."** While definitions of mechanism can vary across levels of analysis, I think causality is difficult to claim without perturbation or stronger experimental discrimination between alternative drivers. In my view, the term is acceptable only in the limited sense of a **bottom-up process model**. Because the causal environmental cue has not been identified directly, and because the center-attraction term is inferred on parsimonious grounds rather than demonstrated explicitly, the model may be better framed as a **sufficiency model** than as a fully established biological mechanism. I think this emphasis on **parsimony** and **sufficiency** could also be reflected more clearly in the Abstract.
