# Supplementary material for: The complex swarming dynamics of malaria mosquitoes emerge from simple minimally-interactive behavioral rules
Source: PLoS Comput Biol. 2026 Aug 26;22(8):e1014685. doi: 10.1371/journal.pcbi.1014685 (PMC13529247; doi:10.1371/journal.pcbi.1014685)
Supplement: Supplementary file 4 [file pcbi.1014685.s014.pdf]

Editor comments:

Comment 0.1: While two of the reviewers are now mostly satisfied with the revisions, reviewer #2's major concerns remain largely unresolved. Here, we tend to side with reviewer #2. In particular, we agree that:

- Artifacts caused by the experimental setup (e.g., cage geometry) cannot be ruled out based on the presented data. Proper controls for this would be essential. Ultimately, the conclusions based on the current data are too strong.

- The dynamic range of swarm sizes in the experiments is insufficient to support the current conclusions.

Resolving these issues will require either additional experimental evidence or drastic changes to the main conclusions (see the specific suggestions of reviewer #2). The latter option would require changes that go beyond what can be done in a major revision and would fundamentally alter the manuscript.

If you believe you can address these concerns, we are willing to consider a substantially revised manuscript as a new submission.

Reply 0.1: We thank the editor for the careful handling of our manuscript and for providing the opportunity to resubmit a substantially revised version for renewed consideration. As noted in the decision letter, two issues raised by reviewer #2 required major revision: (1) potential artefacts arising from the experimental setup, and (2) the limited dynamic range of swarm sizes represented in our original dataset.

To address the first point, we extended our computational and modelling analysis beyond the (Poda et al., 2024) dataset by incorporating a second, independent swarming-flight dataset (Feugère et al., 2022), and rewrote a significant part of the manuscript. This allowed us to evaluate the robustness of our findings across distinct laboratory environments, arena geometries, and marker configurations.

To address the second point, we more clearly discussed the effect of swarm size on swarming dynamics within the available datasets and compared the resulting size range with that reported from natural *Anopheles* mating swarms. This places both the validity and the limitations of our conclusions clearly within their ecological context.

Finally, we added a dedicated limitations section to the revised discussion, in which we explicitly describe the constraints of our study, most notably, the differences between laboratory and natural swarms, thereby directly addressing the core concerns raised by the reviewers.

Please find below our point-by-point responses to all comments of the three reviewers.

Review comments and replies

Reviewer: 1

Comment 1.1: I thank the authors for substantially improving the manuscript. The revisions clarify the conceptual framework and highlight the manuscript's contributions to the literature much better.

I find the addition of individual-trajectory comparisons highly useful in demonstrating that the swarming behavior is scale-free. While I do not entirely agree that the absence of group-size effects conclusively indicates little or no contribution of social cues, the authors are entitled to argue for parsimony (i.e., that environmental cues alone drive this behavior) given the breadth of analysis presented.

Echoing another reviewer's comments, I believe further work is needed to elucidate the precise relationship between environmental factors and swarming dynamics. I therefore look forward to the authors' future contributions on this topic.

Reply 1.1: We thank the reviewer for their positive and constructive assessment of the revised manuscript, which really helped improve both the clarity and positioning of the study.

Reviewer: 2

Comment 2.1: Without additional experiments, the conclusions of this work, particularly the claim of mosquito alignment with the sunset direction, are not sufficiently supported by the data. Therefore, I do not recommend publication of this work in its current version. Below, I provide comments on the authors' responses. To clearly distinguish my remarks from those of the authors, each paragraph is prefaced with "Reviewer" or "Authors", respectively.

Reply 2.1: We thank the reviewer for raising concerns about the strength of the empirical support, especially regarding the interpretation of directional alignment. We agree that additional targeted experiments would provide valuable insight. However, such experiments fall outside the scope of our computational and modelling-focused study.

To address this concern without collecting new data, we added a second, independently collected and peer-reviewed dataset to the study (Feugère et al., 2022). This dataset was obtained under substantially different conditions, including a larger and more open swarming arena. Despite these differences, it shows highly similar swarm structure, turning behavior, and directional tendencies (Figs. 2-3 and S12–S15). This cross-dataset comparison strengthens confidence in the empirical patterns used to parameterize our model and reduces the likelihood that these patterns are artefacts of a single experimental setup.

To incorporate this new Feugère dataset into the study, we performed a complete series of new analyses, added the new resulting figures to the supplement (S12-S15), and rewrote large parts of the Methods, results and discussion sections.

Moreover, in the revised manuscript, we now state explicitly that directional alignment should be interpreted as a consistent empirical pattern rather than as definitive evidence for a specific environmental driver. Establishing causality, for example testing whether sunset cues directly induce alignment, will require new experimental manipulations. We have added a dedicated limitations section to the manuscript where we describe this point clearly and outline which future experiments are needed (lines 619-696).

In the revised manuscript, we also clarify that behavioral rules may differ across species and environmental contexts, and that the minimal rule set we identify is not intended as a complete description of all possible interactions (lines 650-660). The primary aim of our study is to test whether simple interaction rules can generate realistic swarm-level dynamics. Both our original analyses and the new cross-dataset validation support this conclusion.

We thank the reviewer for the feedback. It stimulated us to add the second dataset, which has clearly strengthened the generality and robustness of the manuscript.

Comment 2.2: Authors: Reply 2.2: We thank the reviewer for raising this important point. We agree that the observed flight direction and saccadic maneuvers could, in principle, be influenced by the physical constraints of the flight arena. To address this, we performed a dedicated analysis quantifying the proximity of mosquitoes to the arena walls during saccadic turns (see new Fig. S11).

Reviewer: The distance from the wall at which saccades occur is indeed an interesting quantity; however, the analysis presented here cannot objectively exclude the possibility that the cage interferes with mosquito flight dynamics.

In Figure S11, authors show the distributions of these distances along the two axes x and y, and in the discussion they focus on mean values. I see two weaknesses in this approach. First, the most informative part of these two distributions is not the mean but the left tail, which measures the shortest distances from the wall. Second, as discussed in detail below, the comparison with the distance of collision avoidance is not motivated to assess whether there is an influence of walls on mosquitoes dynamics.

The two histograms shown in Fig.S11 show that the distributions are quite different between the two axes. Along the x-axis the left tail indicates that mosquitoes do not get closer than 10/15cm to the wall. Along the y-axis, instead, saccades are found at very short distances from the walls, where it is unlikely that the effects of confinement are negligible. A substantial fraction of saccades is also found in the region (highlighted in red) that defines the distance of the swarm from the swarming arena. These histograms would be conclusive only if the left tails were comparable along both axes, indicating similar wall-related behavior in the x and y directions.

We cannot tell from this data, whether this is a genuine effect of the alignment with the light, or instead it is the effect of the confinement. The only way to discriminate between these two potential interpretations is to perform experiments changing the position of the light source and checking swarming behavior under these different conditions.

Reply 2.2: We agree with the reviewer that if the goal were to study potential mosquito-wall interactions, the most informative part of the distribution would indeed be the left tail of the distance-to-wall histograms (Fig. S11). However, our objective here is different: we aim to show that mosquitoes typically perform saccadic maneuvers far from the arena boundaries. Because our analysis focuses on average flight dynamics, this suggests that wall effects are unlikely to have significantly influenced our conclusions.

The reviewer further argues that the analysis would only be conclusive if the left tails of the distributions along the two arena axes were comparable. We respectfully disagree. Comparable left tails could instead indicate that mosquito behavior is potentially similarly constrained by the walls in both directions. In contrast, the strong asymmetry observed here suggests that mosquitoes were at least not constrained along the x-axis (the longer dimension of the arena). Moreover, if mosquitoes were strongly constrained along the y-axis, we think it would be difficult to explain why this is also the axis along which they

exhibit the largest displacements. We rewrote and extended discussion about potential wall-effect in the revised discussion, including adding references to documented wall interaction distances of mosquitoes (lines 661-673, (Cribellier et al., 2024; Nakata et al., 2020)).

Additionally, and as outlined in Reply 2.1, we evaluated robustness of our results using the independent Feugère dataset. Under more open boundary conditions, we observe similar localization of saccades, turn kinematics, and directional organization (thorough the manuscript and lines 650-660; Figs. S12-S15). These additional results do not rule out wall effects, but they make it unlikely that the main behavioral patterns arise from a specific enclosure.

Moreover, following the reviewer's recommendation for caution, we now state explicitly that causal mechanisms cannot be inferred from the present data. Only targeted manipulations, such as experimentally moving walls or changing the position and properties of the sunset cue, can identify the relative contributions of boundaries and lighting to orientation and turning behavior. We added this point to a dedicated limitations subsection of the revised manuscript (lines 650-660), and we softened any phrasing that might imply causal claims.

Finally, main objective of our study is to test whether a minimal, biologically plausible rule set can reproduce observed swarm-level structure across independent datasets. The close agreement between the Poda and Feugère datasets strengthens this computational conclusion while leaving causal explanations to future targeted experiments.

Comment 2.3: Authors: Our results show that mosquitoes turned at average distances of 45 ± 8.5 cm from the side walls and 23 ± 7.1 cm from the front/back walls—equivalent to 75 and 38 body lengths, respectively. These distances are an order of magnitude greater than the inter-individual distances that trigger collision avoidance (~2.5 body lengths; Fig. 5K, [8]).

Reviewer: The comparison between the distance from the walls at which saccades occur and the distance that triggers collision avoidance with other insects is not motivated. Such a comparison seems to imply that mosquitoes avoid flying toward a wall using the same mechanism involved in collision avoidance with other insects, which is unlikely. A wall imposes a hard constraint on the mosquitoes' flight phase space, whereas the presence of another nearby insect does not. This argument would have been compelling if the authors could objectively prove that the presence of a wall would influence mosquitoes dynamics in the same way as another insect flying nearby, which is not shown here. Otherwise, the comparison remains quantitative but strongly speculative.

Reply 2.3: We thank the reviewer for this important clarification. First, we fully agree that wall avoidance and collision avoidance between conspecifics rely on different mechanisms. Our original intention was only to provide an order-of-magnitude reference for spatial scale, not to imply that the same sensory processes are involved. In the revised manuscript, we removed any language that could

be interpreted as a mechanistic comparison and now state clearly that the wall-distance observation is circumstantial (lines 661-673). Second, we expanded the manuscript to note that manipulations of boundary geometry or lighting cues are needed to determine how turning behavior is shaped by environmental cues (lines 650-660).

Finally, we like to note that our conclusions on turning dynamics do not depend on the wall-distance scale comparison. They are supported instead by the new cross-dataset analysis using (Feugère et al., 2022), which shows highly similar swarm structure and turn kinematics in a much larger and more open arena (lines 374-394, 457-471 and 488-500, Figs. S12-S15). This strengthens confidence that the key behavioral patterns we analyze are not unique to the Poda recording setup. We thank the reviewer for prompting these clarifications, which helped us present the argument with higher precision and balance.

Comment 2.4: Authors: Additionally, the preferred flight direction—orthogonal to the sunset horizon—aligns with the short axis of the arena (y-axis), which is counterintuitive if wall effects were dominant. If cage geometry were driving the behavior, we would expect alignment along the long axis (x-axis), which is not observed.

Reviewer: This argument is too speculative and not supported by the data. Intuitiveness or counterintuitiveness cannot be used to assess the validity of an interpretation, particularly in biology. Therefore, I invite the authors to either revise or remove this part of the manuscript, or to support this conjecture with appropriate references from the literature.

Reply 2.4: We agree that intuition is not a sufficient basis for interpreting directional alignment. To provide a more quantitative assessment, we previously added an analysis of the distances between saccadic maneuvers and the arena walls (Fig. S11), showing that most maneuvers occur at distances much larger than those expected if direct wall interactions governed turning behavior (rewritten and expended in lines 661-673). At the same time, we recognize that this analysis cannot completely rule out wall effects entirely.

As also mentioned above, we addressed this limitation by expanding our study by incorporating an independent dataset from (Feugère et al., 2022) (Figs. S12-S15). The agreement between the resulting two datasets offers a stronger basis for interpreting the alignment than our earlier, more speculative argument. We updated the manuscript accordingly (thorough the manuscript, lines 374-394, 457-471 and 488-500, Figs. S12-S15).

Comment 2.5: Authors: Together, these findings strongly suggest that wall avoidance does not explain the observed saccadic maneuvers or flight orientation. Instead, the data support our interpretation that these behaviors are visually mediated interactions with the marker.

We acknowledge the reviewer's suggestion for further experiments to disentangle these effects. While such tests (e.g., varying sunset direction or marker shape) would be valuable for refining our understanding of swarm geometry, they fall outside the main scope of this study. Our primary aim is to identify the behavioral rules underlying mosquito swarming, rather than to characterize the exact shape or orientation of the swarm. Moreover, our newly-added analysis based on current data already provide strong evidence that the observed behaviors are not artifacts of the experimental setup. We have added this new analysis and expanded the discussion accordingly (lines 420-426 and 564-570; Fig. S11).

Reviewer: I do not think that further experiments will be relevant to refining the understanding of swarm geometry. They are necessary to confirm that the preferred direction is related to the light, which is one of the behavioral rules highlighted in the manuscript, and to actually prove that the same behavior is observed under a different setup. If this is not the case, i.e, if authors believe that the fact that the preferred direction of flight is relevant as a result of their study but only saccades are relevant, they should revise the manuscript and remove the claims about the direction of light from the abstract as well as from the result section, leaving it at a pure speculative level in the discussion.

Reply 2.5: We agree that our data cannot establish that mosquitoes use sunset cues for alignment, and we therefore no longer state that sunset light is the causal driver. We describe the observation neutrally as alignment along the sunset axis (y-axis pointing toward the sunset), and note that several environmental cues may contribute to this orientation (lines 650-660).

That said, the newly added (Feugère et al., 2022) dataset shows emergence of the same alignment pattern as we found in our original study, despite its substantially larger and more open flight arena. This consistency indicates that the alignment is not specific to the geometry or lighting of a single setup, and therefore represents a reproducible empirical pattern. At the same time, the combined datasets still do not definitely identify the underlying cue, and alternative explanations remain possible.

Determining the causal mechanism, whether sunset light or another cue, will require additional experiments with targeted manipulations of lighting, marker cues, or other environmental features. We now state this explicitly in the newly-added limitations section of the discussion (lines 650-660). Our primary aim remains to test whether a minimal, environmentally driven rule set reproduces swarm-level patterns, independent of the precise cause of directional alignment. We hope the reviewer agrees that these revisions provide a more balanced and appropriately cautious interpretation of the alignment behavior.

Comment 2.6: Authors: Reply 2.3: We appreciate the reviewer's insightful comment. Indeed, distinguishing between attraction to conspecifics and attraction to an environmental marker is challenging in collective behavior studies.

Two key findings support that the marker is the primary swarm attractor:

1. Stable swarm position: Swarms consistently remained above the marker for extended periods. If attraction were primarily to conspecifics, we would expect positional drift over time—especially given that our chamber design allows for such movement. This drift was never observed. 2. Swarming by single individuals: We recorded flight kinematics from the onset of swarming (single mosquito) to fully developed swarms (up to 26 individuals; Fig. S8). Even solitary males exhibited sustained swarming above the marker, with kinematics comparable to those in larger swarms (see Reply 1.5 for details). This suggests that attraction to the marker is independent of swarm size. Based on these results, attraction to the marker remains the most parsimonious explanation for swarm cohesion. To test this, we developed an agent-based model using the marker as the sole attractor, with collision avoidance as the only conspecific interaction. This minimal model successfully reproduced swarm kinematics across group sizes, indicating that explicit attraction between individuals is not required. While we cannot entirely exclude conspecific attraction, our data show it is not necessary to explain mosquito swarming. We have updated the manuscript to include these analyses and expanded the discussion on the model's assumptions and limitations (lines 429-437 and 537-549).

Reviewer: I agree that the attraction to the marker is the most parsimonious choice. However, I would not use the argument of a single mosquito flying on the marker to assess its validity. The crucial role of environmental markers in insect swarms is already well established in the literature, and inter-individual interactions should be viewed not as an alternative to marker attraction, but as an additional ingredient. The absence of positional drift, or the ability of a single individual to remain above the marker, does not discriminate between an underlying interaction driving swarming behavior and a response to external cues; by this logic, the present work, as well as much of the existing swarming literature, would not be needed.

Despite this remark, if the authors wanted only to show that stable swarms can be reproduced with very simple rules, I think the manuscript would benefit from a clearer comparison with existing models. For example, models developed in Ouellette's lab are able to reproduce stable swarms without explicitly incorporating inter-individual interactions. In this context, it is not clear in what sense the present model represents an advance over the current state of the art.

Reply 2.6: We agree with the reviewer that attraction to environmental markers is well established, and we do not claim novelty on this point. The models referred to by the reviewer provide valuable complementary perspectives on swarm aggregation, but they address different mechanistic hypotheses than the one investigated here. Our work focuses on identifying a minimal set of biologically plausible sensory-motor rules at the individual level and testing whether these rules are sufficient to reproduce the observed swarm structure and kinematics in the available empirical datasets. The advance of the present work therefore does not lie in generating stable swarms per se, but in demonstrating that detailed empirical patterns in mosquito swarms can emerge from an explicitly defined minimal rule set.

To make this clear, we revised the manuscript to better distinguish our approach from existing models. In particular, we added a dedicated paragraph in the discussion outlining how our bottom-up mechanistic model is complementary to top-down approaches based on effective forces or statistical inference, including the models developed in Ouellette's lab (lines 620-631). This new section explains that statistical and effective-force models quantify group-level patterns, whereas our model tests whether such patterns can arise from explicit, minimal behavioral rules.

Finally, while we cannot rule out additional conspecific interactions, our analysis shows that these are not required to reproduce the observed swarm dynamics within the swarm-size range present in the datasets. We updated the discussion to clarify the assumptions and limitations of the model accordingly (lines 619-696).

Comment 2.7: Authors: Reply 2.4: As requested by the reviewer, we now clarify in detail the number of swarming experiments performed (six swarms with three recordings per swarm), and the number of swarming mosquitoes per swarm (from 1 to 26 individuals) thorough the manuscript. Moreover, we now explicitly state the sample sizes used for each figure in its figure legend. We also discussed how the number of swarming mosquitoes did not seem to affect their swarming behavior (lines 537-549).

Reviewer: I thank the authors for adding details on the number of swarming experiments. However, I would use much more caution, all over the manuscript, in claiming that the swarm size does not seem to affect swarming behavior, since the data presented here refer to a quite limited size (from 1 to 26 mosquitoes), in particular when compared to field swarms, which may be composed of thousands of individuals, as shown in [1]. In this regard, the section "Variations in flight kinematics with swarm size" (lines 419-437) should be revised or removed.

[1] Ouédraogo TFX, Sawadogo SP, Millogo AA, Niang A, Ouedraogo J, Ouattara SB, Cribellier A, Namountougou M, Dabiré RK, Muijres FT, Diabaté A. Characterization of factors influencing swarm dynamics and mating efficiency in Anopheles coluzzii. Parasit Vectors. 2025 Nov 27. doi: 10.1186/s13071-025-07151-w. Epub ahead of print. PMID: 41310880.

Reply 2.7: We appreciate the reviewer's reminder to place our swarm sizes in their ecological context. Field studies report broad, right-skewed size distributions, with the most frequently observed and median swarm sizes typically in the range of a few to a few dozen males (Assogba et al., 2014; Gueye et al., 2023; Kaindoa et al., 2019). The laboratory swarms we analyzed therefore overlap with the sizes most commonly encountered in nature (lines 674-680).

That said, our datasets do not include very large field swarms that do regularly occur in nature, and claims should not extend beyond the observed range. In response, we tempered wording throughout the manuscript and explicitly limited our conclusions to small-to-medium swarm sizes. In addition, we added a dedicated limitations section to the revised discussion, where we explicitly state that our findings

should not be extrapolated to very large swarms (lines 680-685). Here, we also note that testing model performance across such large swarms will require dedicated field-kinematics datasets, which are currently unavailable, and we highlight this as an important direction for future work.

Comment 2.8: Authors: Reply 2.5: As requested by the reviewer, we extended the discussion section in the revised manuscript. Hereby, we including a comparison of the studied mosquito swarming with other group behaviors (lines 599-612), we revising the paragraph discussing how our study informs us on male-female interactions in mosquito mating swarms (lines 590-598), and we expended the part of the discussion about vector control (lines 613-625). We hope that the reviewer is satisfied with the extended discussion section.

Reviewer: In general, I am not particularly in favor of general discussions that are not directly related to the results. I appreciate the authors' effort to broaden the discussion of the relevance of their work, but I do not consider it necessary, nor do I think it adds anything substantial to the main message of the manuscript. The relevance of this study seems primarily related to fundamental biology, which is as valuable as applied biology, and I would prefer a discussion oriented at fundamental biology.
Reply 2.8: We appreciate the reviewer's perspective. Our study is indeed primarily relevant to fundamental biology, and the discussion is now structured so that the central focus remains on the mechanistic and behavioral insights arising from our computational analysis. At the same time, because *Anopheles coluzzii* is a major malaria vector, even fundamental advances in understanding male swarming behavior have potential implications for applied research. For that reason, we chose to retain the few concise sentences outlining how our findings may relate to applied contexts. We believe this is appropriate, as it provides broader context without distracting from the main fundamental message of the manuscript. This ensure that the discussion remains centered on the biological mechanisms of swarming, while still acknowledging the wider relevance of this malaria vector.

Comment 2.9: Authors: Reply 2.6: As mentioned in our Reply 2.2, we have added a dedicated analysis addressing potential effects of the laboratory environments on the recorded mosquito behaviors. We discuss this in the methods, results and discussion sections of the revised manuscript (lines 420-426 and 561-570; see also Fig. S11).

Reviewer: As already discussed above, I do not think that this analysis is robust enough and does not prove that mosquitoes behavior is not affected by the experimental setup.

Reply 2.9: We agree that any behavioral study must assess how the experimental setup may influence results. This is pertinent here too because the swarming dataset used of our study was recorded under laboratory conditions that differ from the field.

In line with this, comparing our original data with the newly added Feugère dataset provides a direct robustness test under different laboratory constraints (see also replies 2.2 and 2.5). Across both datasets, we observe consistent swarm structure, turning dynamics, and directional alignment, supporting that the behaviors we analyze are reproducible across recording environments rather than artefacts of a single enclosure. At the same time, both datasets are still laboratory recordings. Comparisons with semi-field or field data would be the ideal next step, but high-fidelity swarm-kinematics recordings from the (semi)field are not yet available at the required spatial and temporal resolution. We hope that the addition of the Feugère dataset, together with the explicit limitations subsection, makes clear how we addressed setup-related influences and appropriately circumscribed the scope of our conclusions.

Comment 2.10: Authors: Reply 2.5: We have now included the material and methods section in the main text, thus resolving this issue.

Reviewer: I thank the authors for this change.

Reply 2.10: We thank the reviewer for acknowledging this change.

Comment 2.11: Authors: Reply 2.6: We believe that the reviewer refers to results presented in Fig. S4. In that figure, we compare the 2D heat maps of mean angular speed or of saccade-to-straight flight ratio in the XY and YZ plans. When comparing the heatmaps from the experimental data and from simulated data, we see that both real and simulated mosquitoes have on average higher angular speeds on the boundaries of the swarm, thus showing high proportion of saccadic maneuvers at the edges, and high proportion of straight flights in the middle of the swarm. These 2D distributions of both experimental and simulated data follow a clear axis symmetry around the central vertical axis of the swarm (Fig. S4). Therefore, it is unclear to us which asymmetry the reviewer refers to.

There are, however, key differences between the distributions of the experimental data and simulated data. Most importantly, in the simulations we observe high angular speed and high proportion of saccades around the swarm boundaries (Fig. S4c,d and Fi. S4g,h). This is due to a simplification of our agent-based model where mosquitoes will only trigger a saccade if they are going to exactly cross the swarm boundaries (i.e. binary threshold), while in reality they can trigger a saccade a bit before or after crossing the boundary. This results for the experimental swarms in more diffused distributions of saccades in space (Fig. S4c,d and Fi. S4g,h) and less defined swarm boundaries (as visible on Fig. 5C,D). We have now included a comparison of the experimental and simulation results in the discussion (lines 520-536 and 571-578), highlighting these similarities and differences

Reviewer: I apologize for not having specified that I was referring to Figure S4, as correctly guessed by the authors. In Figure S4 (panels b and d), the authors show the distributions on the xy-plane of the

angular speed and of the saccade-to-straight flight ratio. In both plots, I see an asymmetry between x and y directions, which does not seem to be in agreement with the symmetry around the central vertical axis claimed by the authors.

As stated by the authors, both real and simulated mosquitoes have on average higher angular speeds on the boundaries of the swarm, thus showing a high proportion of saccadic maneuvers at the edges, and high proportion of straight flights in the middle of the swarm. However, the experimental distributions also show a clear axis dependence: at the edges of the swarm along the y-direction, mosquitoes reach higher angular speed than those at the edges of the swarm along the x-direction. Similarly, the saccade-to-straight flight ratio measured at the swarm boundaries exhibits a directional dependence.

This asymmetry is not present in the simulations. This discrepancy is not discussed in the manuscript, and I do not think it may be due to the binary threshold for the boundaries used in the simulations.

Reply 2.11: As pointed out previously, our agent-based model is intentionally minimal and designed to capture the core mechanisms of swarm cohesion, namely, attraction to a swarm center and boundary-mediated turning. Thus, when comparing simulations and experiments, the dominant differences in Fig. S4 are explained by the simplified (binary) boundary condition implemented in the model, which produces sharper spatial localization of saccades relative to the more diffuse experimental distributions. We agree with the reviewer that a residual discrepancy remain in the directional asymmetry highlighted here (note that this discrepancy doesn't exist in the Feugère dataset (Fig. S12a,c)). Explaining such small discrepancies is beyond the scope of the present study, and we therefore suggest retaining the minimal model for its explanatory value regarding the sufficiency of simple interaction rules.

Finally, we thank the reviewer for their dedicated and detailed evaluation of our manuscript and study. Their comments prompted us to scrutinize our analyses, incorporate an additional dataset, and present our conclusions in a clearer and more balanced framework.

Reviewer: 3

Comment 3.1: The authors did a great job addressing my comments. The updated methods are indeed much clearer and the model description enables model reproducibility. The data and code repository is also well structured and easily accessible. I am happy to recommend this article for publication but please find some minor comments on 3 points below:

- I like the additional analysis and text regarding swarm size effects. I was wondering whether density effect can start playing a role in larger aggregations, where there may be more competition for instance for positions closer to the center of the attractor and females. The authors nicely discuss this in lines 537-549 that usually the swarms don't reach higher densities in nature since the volume of the swarm usually also increases, but if there is any additional ecological insight in the literature (on what the males compete for or on female preference [eg. 1]) could be a nice addition here to solidify the argument (or a mention that this gap of research could be addressed with more data in the future). For instance, under the prism of collective behavior [2], why would interactions among males not matter on their mating success?

[1] Ng'habi, K. R., Huho, B. J., Nkwengulila, G., Killeen, G. F., Knols, B. G., & Ferguson, H. M. (2008). Sexual selection in mosquito swarms: may the best man lose?. Animal Behaviour, 76(1), 105-112. https://doi.org/10.1016/j.anbehav.2008.01.01

[2] Rathore, A., Isvaran, K., & Guttal, V. (2023). Lekking as collective behaviour. Philosophical Transactions of the Royal Society B, 378(1874), 20220066. https://doi.org/10.1098/rstb.2022.0066

Reply 3.1: We thank the reviewer for the positive assessment of the manuscript and for recognizing its clarity, reproducibility, and relevance to the journal. We appreciate the thoughtful suggestions aimed at strengthening the ecological and conceptual context of the study. Following the reviewer suggestion, we added more details discussion of these questions as well as the suggested references in the manuscript (lines 610-617).

Comment 3.2: Minor:

Lines 436 ('confirms previous suggestions that a mosquito mating swarm of a single individual can exist [21, 48]') and 541('supports the observation that even a single mosquito can display swarming behavior in the absence of conspecifics [21,48]') are rather overlapping, I think the first one in the results is unnecessary and can be omitted (also because a single individual shouldn't be called a swarm, but rather showing swarming behavior as the authors write later).

Reply 3.2: We agree with the reviewer. We removed the redundant phrasing in the results section and retained only the clearer formulation emphasizing that a single individual can display swarming behavior, rather than referring to a “swarm” of one individual.

Comment 3.3:

- Comparisons with other species: I find that the new discussion paragraph nicely puts the results in a wider context (lines 599-612). I only find unclear what the authors mean by framework (line 611) here (a collection of metrics? Or the behavioral algorithm of the model?). Perhaps they could add a clarification. A nice link here could also be with a recently developed framework to compare collective motion across species (‘swarm verse’ [3]) that has been focused only on vertebrates. Mating swarms of insects seems like an ideal space to adjust this framework to, expanding towards invertebrates.

[3] Papadopoulou, Marina, Simon Garnier, and Andrew J. King. "swaRmverse: An R package for the comparative analysis of collective motion." Methods in Ecology and Evolution 16.1 (2025): 29-39. https://doi.org/10.1111/2041-210X.14460

Reply 3.3: By “framework” we refer to the statistical approach from (Iacomelli et al., 2025) mentioned earlier in the text. We clarified this explicitly in the text by changing the word “framework” by “approach”, and by referring to the study again (lines 624-630). We also agree that recent comparative approaches such as the “swaRmverse” framework are relevant and we now cite and mention how mosquito mating swarms could extend such comparative efforts to invertebrate systems (lines 700).

Comment 3.4:

- In the author summary, the sentence: ‘how male malaria mosquitoes form mating swarms’ may make readers think about long range attraction to environmental cues and one another to ‘initiate’ a swarm rather than how the swarming characteristics arise. Perhaps the authors should revise/clarify this.

Reply 3.4: We agree with the reviewer’s concern and have revised the author summary to clarify that our study focuses on how swarming kinematics and structure arise once swarming is initiated, rather than on long-range attraction mechanisms or swarm initiation (lines 42).

References

Assogba, B. S., Djogbénou, L., Saizonou, J., Diabaté, A., Dabiré, R. K., Moiroux, N., Gilles, J. R. L., Makoutodé, M., & Baldet, T. (2014). Characterization of swarming and mating behaviour between Anopheles coluzzii and Anopheles melas in a sympatry area of Benin. *Acta Tropica*, *132*(1), S53–S63. https://doi.org/10.1016/J.ACTATROPICA.2013.09.006

Cribellier, A., Camilo, L. H., Goyal, P., & Muijres, F. T. (2024). Mosquitoes escape looming threats by actively flying with the bow wave induced by the attacker. *Current Biology*, *34*(6), 1194-1205.e7. https://doi.org/10.1016/j.cub.2024.01.066

Feugère, L., Roux, O., & Gibson, G. (2022). Behavioural analysis of swarming mosquitoes reveals high hearing sensitivity in Anopheles coluzzii. *Journal of Experimental Biology*, *225*(5), 243535. https://doi.org/10.1242/JEB.243535/VIDEO-1

Gueye, O. K., Niang, A., Faye, M. B., Dia, A. K., Ahmed, A. A., Sy, O., Tripet, F., Konaté, L., Dia, I., Wondji, C. S., Gaye, O., Faye, O., Diabate, A., & Niang, E. H. A. (2023). Characterization of the swarming behavior of Anopheles coluzzii and Anopheles gambiae (Diptera: Culicidae) populations in a hybrid zone of Senegal. *Journal of Medical Entomology*, *60*(6), 1278–1287. https://doi.org/10.1093/JME/TJAD120

Iacomelli, G., Lombardi, M., Parisi, L., Fiorini, M., Grucci, F., Lavorgna, A., Ligato, M., Zarcone, G., Peirce, M. J., Melillo, S., & Spaccapelo, R. (2025). Experimental evidence of male-male interaction in laboratory swarms of Anopheles gambiae mosquitoes. *BioRxiv*, 2025.11.06.686941. https://doi.org/10.1101/2025.11.06.686941

Kaindoa, E. W., Ngowo, H. S., Limwagu, A. J., Tchouakui, M., Hape, E., Abbasi, S., Kihonda, J., Mmbando, A. S., Njalambaha, R. M., Mkandawile, G., Bwanary, H., Coetzee, M., & Okumu, F. O. (2019). Swarms of the malaria vector Anopheles funestus in Tanzania. *Malaria Journal*, *18*(1), 29-. https://doi.org/10.1186/S12936-019-2660-Y/FIGURES/6

Nakata, T., Phillips, N., Simões, P., Russell, I. J., Cheney, J. A., Walker, S. M., & Bomphrey, R. J. (2020). Aerodynamic imaging by mosquitoes inspires a surface detector for autonomous flying vehicles. *Science (New York, N.Y.)*, *368*(6491), 634–637. https://doi.org/10.1126/science.aaz9634

Poda, B. S., Cribellier, A., Feugère, L., Fatou, M., Nignan, C., Hien, D. F. de S., Müller, P., Gnankiné, O., Dabiré, R. K., Diabaté, A., Muijres, F. T., & Roux, O. (2024). Spatial and temporal characteristics of laboratory-induced Anopheles coluzzii swarms: Shape, structure, and flight kinematics. *IScience*, *27*(11), 111164. https://doi.org/10.1016/J.ISCI.2024.111164
