# Supplementary material for: The complex swarming dynamics of malaria mosquitoes emerge from simple minimally-interactive behavioral rules
Source: PLoS Comput Biol. 2026 Aug 26;22(8):e1014685. doi: 10.1371/journal.pcbi.1014685 (PMC13529247; doi:10.1371/journal.pcbi.1014685)
Supplement: Supplementary file 5 [file pcbi.1014685.s015.pdf]

Editor comments:

Comment 0.1: The reviewers have raised several issues that still need to be addressed. In particular, please address Reviewer #2's concerns about the compatibility of the behavioral rules with the new dataset. Additionally, ensure that the conclusions are not overstated, particularly in the abstract and summary sections of the main text.

Reply 0.1: We thank the editor for this summary of the remaining concerns. We have revised the manuscript to avoid overstating our conclusions in the abstract and author summary, and we now more clearly frame our model as a parsimonious explanatory model rather than a fully predictive model across all experimental and natural contexts. We also address reviewer #2's concerns in detail below, especially regarding the compatibility of the behavioral rules with the Feugère dataset.

Please find below our point-by-point responses to all comments.

Review comments and replies

Reviewer: 1

Comment 1.1: The addition of **Feugère et al. (2022)** substantially reduces the concern that the observed swarm shape and organization are artifacts of a single arena geometry. Given the clear differences between the two setups, I think this new comparison removes most of the doubt that the main swarm features arise solely from the specific chamber used in Poda et al. In that sense, the added dataset is a meaningful and welcome strengthening of the manuscript Nevertheless, revisiting the experimental design, I still find that one crucial aspect is missing. The very strong contrast of the dark swarm marker in a confined laboratory setting may not be fully ecologically representative. In this setup, there is effectively a single salient dark landmark available. In the field, by contrast, males may encounter multiple candidate landmarks, and under such conditions social recruitment or other collective effects could still play a role in determining where and how swarms form. Thus, while the present revision is clearly stronger, I think the authors have demonstrated reproducibility of similar mosquito behavior in **two different laboratory** contexts, rather than having fully excluded experimental-context effects more generally.

Reply 1.1: We thank the reviewer for their positive assessment of the revised manuscript, and for highlighting this remaining limitation.

We agree that our study concerns mosquito swarming above single, highly contrasting laboratory markers, whereas natural swarm markers can be more diverse, less contrasted, and potentially occur together with other landmarks. We therefore clarified in the manuscript that our results are based on laboratory datasets, in both the abstract and main text (lines 21, 24, 30, 45, 50, 102, 384, and 455). We also now discuss that these results should be confirmed in more realistic environments (lines 662-663), and using markers with various characteristics such as shape, size and contrast (lines 585-587).

Comment 1.2: While the authors did not perform additional experiments expanding the swarm-size range, I find their revised argumentation acceptable. They now acknowledge much more clearly the ecological range they are probing, and I think that is the correct way to handle this limitation.

More broadly, I think studying low- to mid-density swarms is valuable in its own right, not only because these may correspond to common field swarm sizes, but also because such events can represent the initial stage of larger and more persistent swarm formation. It is also entirely plausible that the effective rules differ in much larger swarms, where physical crowding, density-dependent effects, or other interactions could become more important.

Reply 1.2: We thank the reviewer for this positive assessment. We agree that low to mid-sized swarms are valuable to study, both as ecologically relevant swarms and as possible early stages of larger swarm formation. We also agree that additional processes may become important in larger swarms, and we have kept the interpretation of our results limited to the swarm size range covered by the analyzed datasets. We state this explicitly at several locations in the revised manuscript (lines 174-175, 503, and 702-707).

Comment 1.3: Revisiting the paper, however I still find one point missing. The authors document in **Fig. S8** that, within the **Poda dataset,** the number of swarming mosquitoes ranged from **1 to 26**, and that across this sampled range they detected no clear effect of group size on several **individual flight-kinematics metrics**, namely **saccadic turn angle, straight-flight distance between successive saccades**, and **straight-flight duration.** This is useful and should be stated clearly as a bounded result: within the sampled low- to mid-density range, the measured individual kinematics appear largely insensitive to swarm size. However, the manuscript does not provide an analogous quantification for **swarming duration**, i.e. neither a direct distribution of **full swarm lifetime** nor of **individual residence time** within the swarm. In other words, even if turn angle or straight-flight statistics are unchanged, swarm size could still matter at the level of how long individuals keep swarming and how long the swarm itself remains coherent. Quantifying these persistence-related measures would therefore provide a more complete test of whether swarm size truly has limited influence within the studied range.

Reply 1.3: We agree with the reviewer that quantifying how individual persistence in the swarm relates to swarm size would be valuable. However, we cannot estimate this reliably from the current datasets, because maintaining individual identity for periods above a minute remains difficult, especially when several mosquitoes overlap in position. As a result, track length may be artificially shorter in larger swarms because of tracking limitations rather than biological effects.

Sadly, we also cannot robustly test how swarm size affects full swarm duration. In both analyzed datasets, swarm size changes continuously over time, and the maximum swarm size varies little between replicates, with peak sizes around ~20 mosquitoes. These datasets therefore provide too little variation in average swarm size per replicate to test its effect on swarm duration.

We have now explicitly included these constraints as a fourth limitation in the revised discussion (lines 71-721), where we clarify that our conclusions are restricted to instantaneous flight kinematics and do not address persistence-related dynamics.

Comment 1.4: In general, I also appreciate that the authors are sincere in acknowledging the limitations of the study. However, this caution is still expressed mainly in the Discussion, whereas the more prominent parts of the manuscript remain somewhat stronger in tone. For example, I think the Abstract

could state the swarm-size scope even more explicitly, for instance emphasizing that they studied low-mid densities.

Reply 1.4: We have edited the abstract and author summary to better acknowledge the scope and limitations of the study. In particular, we clarified that our results concern small to medium-sized swarms recorded under laboratory conditions, in both the revised abstract and discussion section (lines 26, 506, 669 and 705).

Comment 1.5: A further minor but important point concerns the repeated use of the term "mechanistic." While definitions of mechanism can vary across levels of analysis, I think causality is difficult to claim without perturbation or stronger experimental discrimination between alternative drivers. In my view, the term is acceptable only in the limited sense of a bottom-up process model. Because the causal environmental cue has not been identified directly, and because the center-attraction term is inferred on parsimonious grounds rather than demonstrated explicitly, the model may be better framed as a sufficiency model than as a fully established biological mechanism. I think this emphasis on parsimony and sufficiency could also be reflected more clearly in the Abstract.

Reply 1.5: We agree with the reviewer that we cannot claim to have identified the exact biological mechanisms of male mosquito swarming, and that doing so would require further experiments, for example using perturbations. We therefore replaced mentions of a "bottom-up mechanistic approach" with "bottom-up process approach" (lines 106 and 646), and removed or rephrased mentions of a "mechanistic model" or "mechanistic basis" (lines 673, 745 and 763). We also edited the abstract and author summary to emphasize that our model provides a parsimonious explanation of the observed swarming patterns (lines 29, 35, and 46-47).

Reviewer: 2

Comment 2.1: I really appreciate the effort made by the authors to expand the analyzed data and extend the discussion.

Reply 2.1: We thank the reviewer for recognizing the additional analyses and the expanded discussion. We appreciate the reviewer's careful reading of the revised manuscript, and address the remaining concerns point by point below.

Comment 2.2: However, I have some concerns regarding the compatibility of the Feugere male dataset with the model introduced in the present work. At this stage, I am unable to recommend publication of the manuscript.

For a model to be meaningful, it must demonstrate predictive power on datasets not used to fit the parameters. Given the availability of multiple datasets, the authors could have followed a standard cross-validation procedure: using the Poda dataset to fit the model parameters, as they do, and then quantitatively assessing the extent to which the Faugere datasets reproduce the predicted behavior. This step is, in my view, not properly addressed and it lacks a quantitative analysis.

Reply 2.2: We thank the reviewer for raising this important point regarding model validation and predictive power.

We agree that, in many contexts, demonstrating predictive performance on an independent dataset is a key step in establishing a model as broadly predictive. In particular, cross-validation between datasets is essential when the goal is to develop a quantitatively predictive model that generalizes across experimental conditions.

In the present study, however, our primary objective is different. We adopt a bottom-up, process-based approach to test whether a minimal set of behavioral rules is sufficient to reproduce the main statistical and spatial features of mosquito swarms. In this context, the model is designed as an explanatory or sufficiency model, rather than a predictive model. The central question is therefore not whether the model can predict an independent dataset without modification, but whether complex swarm structure can emerge from simple, minimally interactive rules grounded in empirical kinematics.

We agree that evaluating predictive performance across datasets would be highly valuable as a next step. However, this is not straightforward here because the Poda and Feugère datasets differ substantially in key environmental features, most notably in marker geometry. Feugère et al. (2022) used an elevated, cylindrical, and asymmetric marker, whereas the Poda dataset was obtained using a flat horizontal square marker. These differences lead to measurable shifts in swarm position and geometry (Fig. S12), indicating that the mapping between environmental input (marker structure) and behavioral response is

not preserved across datasets. As a consequence, the visual boundary rule defined for the Poda dataset cannot be directly transferred to the Feugère dataset without reformulating how marker perception is modeled.

For this reason, we use the Feugère dataset as an independent comparison to assess whether the core kinematic patterns that underpin our model (e.g. alternation between straight flight and saccades, spatial localization of turning, and directional bias along the sunset axis) are robust across experimental conditions, rather than as a strict predictive validation dataset.

We have clarified this point throughout the manuscript and now explicitly distinguish between (i) model evaluation against the Poda dataset, and (ii) qualitative and statistical comparison with the Feugère dataset. We also emphasize that establishing predictive generality across different environmental geometries will require dedicated experiments in which key features such as marker shape and placement are systematically varied (lines 384-388, 661-663, and 723-727).

Comment 2.3: More specifically, my concerns regarding the compatibility of the model with the Feugere male dataset involve both Behavioral rule 1 and Behavioral rule 2, as detailed below.

Behavioral rule 1: Swarming mosquitoes preferentially fly straight through the swarm center, where the marker viewing angle is maximal. They then continue flying straight, until the marker viewing angle falls below a threshold value, which triggers a turning maneuver.

Behavioral rule 1 implies that the swarm center corresponds to the marker center, and that there is symmetry in the structure of the swarm with respect to the marker position. Although the Feugere male dataset exhibits a structural symmetry around its own vertical axis, it is shifted in the y-axis towards the negative direction, see Fig.S12a-b. As a result, part of the swarm is consistently out of the marker, and mosquitoes do not appear to show a preference for the region where the viewing angle is maximized, namely the center of the marker. This discrepancy suggests that behavioral rule 1 fails to predict and reproduce the position of the Feugere male dataset, and therefore highlights the lack of generality of the model. Maximizing the viewing angle of the marker may therefore not be the mechanism underlying swarm formation and spatial structure.

Reply 2.3: We thank the reviewer for highlighting this point. We believe that the shift of the Feugère male swarm along the y-axis is likely related to the asymmetric elevated marker used by Feugère et al (2022). In our model, we used the viewing angle of the square marker along the x-axis because this provided a parsimonious explanation for the Poda dataset. However, we recognize that this rule does not yet explain how mosquitoes respond to more complex swarm markers, such as the elevated marker used by Feugère et al (2022). We have therefore clarified that our current model captures the main spatial features of swarms above a simple symmetric marker, but does not yet provide a general visual rule for all marker geometries (caption of Fig. S12). This limitation does not invalidate the model for the Poda

dataset, but it does show that further work is needed to generalize the visual boundary rule (as now mentioned lines 579-590).

Comment 2.4: Behavioral rule 2. The turning maneuver consists of a rapid saccadic turn within the horizontal plane, with biases towards the swarm center and along the sunset axis (towards and away from the sunset position) [32]. After the turn, the mosquito continues to fly in a straight line (rule 1).

In my opinion this is the most critical aspect. There is a lack of quantitative assessment of the existence of a preferred direction of flight, and authors rely only on a qualitative evaluation of the plots shown in Fig.S13g and Fig.S15f. In detail, my concerns are the following:

1- In Fig.15f, I do not see clear evidence of a preferred flight direction. The distribution is indeed radially symmetric, in contrast with Fig.S15h (female dataset) and Fig.S4 (Poda dataset)

Reply 2.4: We agree with the reviewer that the male dataset (Fig. S15f) does not show an obvious preferred flight direction by visual inspection alone, in contrast to the clearer structure observed in females (Fig. S15h) and in the Poda dataset (Fig. S4). To quantitatively test for directional bias, we performed a circular statistical analysis of flight azimuths relative to the sunset axis. Specifically, flight headings were expressed relative to the sunset direction (90°) and treated as axial data to account for bidirectional movement toward and away from sunset. We then tested for non-uniform alignment using a Rayleigh test on the axial-transformed angles. This analysis revealed a significant alignment with the sunset axis in all datasets considered, including the male Feugère et al. dataset (all $p < 0.001$). We have revised Fig. 2 and S13 captions and text (lines 236-246, 446-447 and 452) accordingly to explicitly reflect that the directional bias is statistically supported.

Comment 2.5: 2- I am struggling to understand how distributions in Fig.S13g are normalized. By definition, the integral of each distribution should be equal to 1. But the distributions in this panel seem to cover areas that are not comparable with each other. To be more clear, let's focus on the dark red and dark blue distributions. The dark red has a well pronounced peak at -90deg and it is quite narrow (the radii of the distributions are very small except for a very narrow portion close to -90deg). The dark blue is instead much broader, covering the entire [-180deg:180deg] interval with a peak at -90deg. By definition of probability distribution, its peak at -90deg cannot have the same radius of the dark red, as instead it has in Fig.S13g: if both distributions are properly normalized, the broader distribution should exhibit a lower peak. This makes the area of the dark blue much larger than the area of the dark red, giving the misleading impression that the two peaks (the one of the dark red and the one of the dark blue) have similar statistical significance. Authors are probably scaling one of the two distributions.

Reply 2.5: The reviewer is correct to assume that the distributions in Fig. S13g were scaled differently. We did this to make the male distributions more clearly visible despite the stronger female peaks and because the integral of each probability distribution will be equal to 1 in both cases. We apologize for not explaining this clearly. Our intention was not to imply that the peak heights were directly comparable, but to show that male flight directions are more broadly distributed than female flight directions, while still showing a preference for the sunset axis. We have now applied identical scaling to the male and female distributions in Fig. S13, allowing direct comparison of peak magnitude and spread.

Comment 2.6: 3- The distributions in Fig.S13g for the Feugere male dataset are computed separately for y>0 and y<0, as done for all the other datasets. This choice would have been reasonable if the swarm were centered with the marker (see comment above). But in this specific situation y=0 does not correspond to any physically meaningful reference position. The distributions should instead be defined with respect to the actual swarm center. Choosing y=0 as a splitting point, likely causes the presence of a significant portion of the dark blue distribution in the region of positive angles, as well as the imbalance between the dark and light blue distributions. Also note that, because of the color code and of the transparency of the plot, the portion of the dark blue distribution in the positive angles region is barely visible, unless when zooming in the figure. Its visibility could be improved using different colors.

Reply 2.6: We agree with the reviewer. Because the Feugère male swarm is shifted relative to the marker center, splitting the data at y = 0 is not the most meaningful choice for this dataset. We have therefore revised the analysis to show the distributions without splitting them (Fig. S13). We also improved the figure visualization to make the distributions easier to distinguish. This revision does not change the main interpretation, but it provides a more appropriate comparison for the shifted Feugère male swarm.

Comment 2.7: 4- To better assess the presence of a preferred flight direction, it would be useful to include, for the Feugere male dataset, a figure showing the distribution of the azimuth angle between two saccades as a function of mosquito positions, analogous to Fig.3c-d and Fig.S5a-b for the Poda dataset.

Reply 2.7: We followed the reviewer's suggestion and added the requested analysis as Fig. S16. This figure shows the distribution of azimuth angles between successive saccades as a function of mosquito position for the Feugère male dataset, analogous to Fig. 3c-d and Fig. S5a-b for the Poda dataset. The new figure provides a useful spatial visualization of the flight-direction bias. Please also note that we updated panels E and F of Fig. 3, and Fig. S5 to correct a discrepancy. We inconstantly showed azimuth and climb angles from before or from after the saccadic position. We now consistently display post-

saccadic angles across all panels and clarified this in the revised captions (Fig. 3E-H, S5 and S16). This revision does not change our interpretation of the results.

Comment 2.8: 5- It would also be important to introduce a quantitative metric to characterize the degree of alignment in the flight directions, rather than relying solely on qualitative interpretation of the distributions.

Reply 2.8: We agree that a quantitative metric is necessary to support interpretation of flight-direction structure. In fact, this is already provided in Fig. S13g, which quantitatively summarizes the distribution of azimuthal headings between successive saccades across datasets and sexes. In addition, we now complement this descriptive metric with a formal circular statistical test of alignment with the sunset axis (Rayleigh test on axial-transformed flight directions), which provides a direct inferential assessment of directional bias.

This statistical analysis shows significant alignment with the sunset axis across datasets (all $p < 0.001$), including the Feugère male dataset, thereby removing the need to rely on qualitative interpretation of the distributions. We have updated the main text and figure captions accordingly to explicitly reflect this statistical support for directional alignment.

Reviewer: 3

Comment 3.1: I think the authors did a great job addressing my previous comments, I do not have any follow up. In relation to the larger discussion with the comments of Reviewer 2, I wanted to mention the following points:

1) I think the newly analysed published dataset is an excellent addition to the paper that indeed provides further support to the findings.

2) Comment 2.2: I agree with the authors' arguments and I find the extended discussion clear and effective in communicating the response to the concerns of Reviewer 2.

2) Comment 2.3: Inter-individual distances are often used as a unit metric, so I don't find it peculiar or confusing in the manuscript. I see that it may be misleading for an interdisciplinary audience and thus the clarification the authors added is a good improvement.

3) Comment 2.6: I agree with Reviewer 2 in relation to the markers and inter-individual attraction. I think a stronger clarification on the comparison with existing models would be beneficial, and I think the authors did a good job explaining this in their new discussion. As the authors mention, testing minimal individual-level rules necessary to reproduce collective patterns is an invaluable insight from such agent-based models with important theoretical contributions despite the possibility of more complex behaviours of individual organisms.

4) Concerning the wall effects overall, as the authors note at Reply 2.9, experimental setups may always have an effect on behaviour and usually cannot be ruled out, it is the cost of having a more standardised alternative to a study in the field. For instance, wall effects are present in most studies with laboratory experiments in fish schools. I think the authors did the best they could in testing these effects, and the new dataset further supports that their conclusions are not overstated. In my opinion, the new limitations section of the Discussion covers everything quite extensively.

Overall, I think that more cautious interpretation is always a plus, especially when studying a complex multi-agent system (where causality is very hard to prove empirically) and when simulations are used, given other potential underlying rules that may be able to reproduce the same emergent properties. Thus, I find the revised manuscript highly improved and the claims of the authors now well balanced and justified. I am happy to recommend its publication.

Reply 3.1: We thank the reviewer for this detailed and positive assessment of the revised manuscript. We are grateful for the reviewer's support, and for recognizing the value of the additional dataset, the expanded discussion, and the more cautious interpretation of the model.

Comment 3.2: Minor remarks:

- Lines 560, I think there is a typo in the first new sentence. Perhaps a reference is also needed in the first sentence of that paragraph.

Reply 3.2: We added references for this sentence and corrected the typo.

Comment 3.3: - There is some repetition in the new part of the discussion, eg. lines 645 vs lines 655. Perhaps the first short paragraph is not necessary and can be merged with the following 2 for conciseness.

Reply 3.3: We thank the reviewer for pointing this out. We removed the repetition to make this part of the discussion more concise.
