# Supplementary material for: The complex swarming dynamics of malaria mosquitoes emerge from simple minimally-interactive behavioral rules
Source: PLoS Comput Biol. 2026 Aug 26;22(8):e1014685. doi: 10.1371/journal.pcbi.1014685 (PMC13529247; doi:10.1371/journal.pcbi.1014685)
Supplement: Peer review history

# Peer review: Decision Letter 0

**DOI:** 10.1371/journal.pcbi.1014685.r001

**Authors:** Tobias Bollenbach, David Fisher

21 Sep 2025

The complex swarming dynamics of malaria mosquitoes emerges from simple minimally-interactive behavioral rules

PLOS Computational Biology

Dear Dr. Cribellier,

Thank you for submitting your manuscript to PLOS Computational Biology. After careful consideration, we feel that it has merit but does not fully meet PLOS Computational Biology's publication criteria as it currently stands. Therefore, we invite you to submit a revised version of the manuscript that addresses the points raised during the review process.

Please submit your revised manuscript within 60 days Nov 21 2025 11:59PM. If you will need more time than this to complete your revisions, please reply to this message or contact the journal office at ploscompbiol@plos.org. When you're ready to submit your revision, log on to https://www.editorialmanager.com/pcompbiol/ and select the 'Submissions Needing Revision' folder to locate your manuscript file.

Please include the following items when submitting your revised manuscript:

* A rebuttal letter that responds to each point raised by the editor and reviewer(s). You should upload this letter as a separate file labeled 'Response to Reviewers'. This file does not need to include responses to formatting updates and technical items listed in the 'Journal Requirements' section below.

* A marked-up copy of your manuscript that highlights changes made to the original version. You should upload this as a separate file labeled 'Revised Manuscript with Track Changes'.

* An unmarked version of your revised paper without tracked changes. You should upload this as a separate file labeled 'Manuscript'.

If you would like to make changes to your financial disclosure, competing interests statement, or data availability statement, please make these updates within the submission form at the time of resubmission. Guidelines for resubmitting your figure files are available below the reviewer comments at the end of this letter

We look forward to receiving your revised manuscript.

Kind regards,

David Fisher

Guest Editor

PLOS Computational Biology

Tobias Bollenbach

Section Editor

PLOS Computational Biology

**Additional Editor Comments :**

Three expert reviewers have carefully considered your manuscript and returned a range of useful comments and suggestions. Some of these are quite major and directly relate to the interpretation of the experimental data and so what inference we can make from your findings. Since most reviewers agreed the work was potentially of interest, I am willing to suggest “Major revisions”, but the reviewers’ comments must be dealt with through substantial revisions before the MS will be sent back out for review.

In particular, please ensure you:

Be clearer about the source of the data, and when figures are modified versions of elsewhere.

Address concerns about the comparison between leks and other types of collective motion, as it impacts on generality of findings.

Re-order to have Methods following Introduction, and be clear on sample sizes throughout.

Consider the other interpretations for your findings the reviewers suggest, and in particular deal with concerns of reviewer #2 that your results might be an artefact of your design.

Failure to robustly address these and other comments will lead to a desk rejection.

**Journal Requirements:**

1) We ask that a manuscript source file is provided at Revision. Please upload your manuscript file as a .doc, .docx, .rtf or .tex. If you are providing a .tex file, please upload it under the item type u2018LaTeX Source Fileu2019 and leave your .pdf version as the item type u2018Manuscriptu2019.

2) Please provide an Author Summary. This should appear in your manuscript between the Abstract (if applicable) and the Introduction, and should be 150-200 words long. The aim should be to make your findings accessible to a wide audience that includes both scientists and non-scientists. Sample summaries can be found on our website under Submission Guidelines:

https://journals.plos.org/ploscompbiol/s/submission-guidelines#loc-parts-of-a-submission

3) Your manuscript is missing the following sections: Introduction, and Methods.  Please ensure all required sections are present and in the correct order. Make sure section heading levels are clearly indicated in the manuscript text, and limit sub-sections to 3 heading levels. An outline of the required sections can be consulted in our submission guidelines here:

https://journals.plos.org/ploscompbiol/s/submission-guidelines#loc-parts-of-a-submission

4) Please upload all main figures as separate Figure files in .tif or .eps format. For more information about how to convert and format your figure files please see our guidelines:

https://journals.plos.org/ploscompbiol/s/figures

5) We have noticed that you have a list of Supporting Information legends for (Supplementary Movies, Data S1, Data S2, and Code S1) in your manuscript. However, there are no corresponding files uploaded to the submission. Please upload them as separate files with the item type 'Supporting Information'.

6) Please amend your detailed Financial Disclosure statement. This is published with the article. It must therefore be completed in full sentences and contain the exact wording you wish to be published.

1) State what role the funders took in the study. If the funders had no role in your study, please state: "The funders had no role in study design, data collection and analysis, decision to publish, or preparation of the manuscript."

7) Please ensure that the funders and grant numbers match between the Financial Disclosure field and the Funding Information tab in your submission form. Note that the funders must be provided in the same order in both places as well. Currently, "the Institut de Recherche our le Développement and the government of Burkina Faso" are missing from the Financial Disclosure field.

Note: If the reviewer comments include a recommendation to cite specific previously published works, please review and evaluate these publications to determine whether they are relevant and should be cited. There is no requirement to cite these works unless the editor has indicated otherwise.

**Reviewers' comments:**

Reviewer's Responses to Questions

**Comments to the Authors:**

**Please note that one review is uploaded as an attachment.**

Reviewer #1: The review is uploaded as an attachment.

Reviewer #2: I am afraid the manuscript is not ready to be published in Plos Computational Biology.

I do not recommend the publication.

My concerns are mostly on the interpretation of the experimental data, which I believe are overinterpreted, and on the conclusions the authors draw from the comparison with the boids model.

DATA INTERPRETATION

Two main features of mosquito behavior are considered crucial in the manuscript. The first is the preferred direction of flight, orthogonal to the sunset horizon. The second is that mosquitoes perform saccades at the border of the swarm, due to a visual interaction (defined by a specific visual range) with the marker.

My concern is that both features may be artifacts of the experimental setup. The direction orthogonal to the sunset horizon corresponds to the shortest side of the cage. Therefore, an alternative interpretation of the data, different from that of the authors, is that mosquitoes are not completely free to move in that direction and that the cage is affecting the behavior. For instance mosquitoes may be induced to perform fast 180deg turns (saccades), to avoid the wall and redirect towards the central, open area of the cage. With a similar reasoning, mosquitoes at the borders of the swarm may be induced to perform saccades, not because of a visual interaction with the marker, but because of a physical constraint of the cage itself.

To determine which of the two alternatives (the one proposed by the authors and the one described above) is the more realistic, the direction orthogonal to the sunset horizon must be separated from the shortest side of the cage, and the swarm boundaries must be separated from the cage boundaries. These variables cannot be decoupled in the data presented here.

Therefore, authors need to perform additional experiments: 1- with the sunset in a different position/direction to test whether the preferred direction is related to the sunset; 2- with different markers (shape and size) to validate the proposed visual interaction.

BOIDS MODELS

To the best of my knowledge, the dominant term in boids models is the alignment interaction, as in other interacting models, like Vicsek’s. Repulsion and attraction are essentially needed to have realistic simulations and applications in robotics.

The low relevance of repulsion is indeed in line with what the authors find in their data, where repulsion events are rare. In regard to the attraction term, the situation is more subtle. Boids model considers attraction towards the average position of the neighbors. In large systems, this means that particles in the bulk will not feel the effect of the attraction. The attraction is instead dominant for particles at the periphery, and attraction is actually needed to avoid a loss of cohesion.

In the simulation presented in the manuscript, all the particles are essentially at the periphery of the swarm, since there are only 10 particles in the group. As a result, they are all influenced by the attraction toward the average position of the other ones, which due to the limited setup, always coincides with the position of the landmark. It is then not possible to discriminate between the presence of an individual attraction toward the marker, as suggested by the authors, or an attraction toward the other members of the group. This ambiguity should be discussed within the manuscript.

MINOR COMMENTS

1- The number of mosquitoes in the analyzed swarms should be clearly stated. At the beginning of the Experimental section, authors mention that they reconstructed 938 tracks. In SI, section “Experimental conditions”, they describe their experimental procedure and refer to the number of mosquitoes released in the cage (30 or 50 depending on the experiment). It is only in the description of the simulations, SI Section “Agent-based model simulations”, that they finally state that the data in the manuscript refer to swarms of approximately ten mosquitoes. It would be better to clarify from the beginning of the manuscript that the data are 938 reconstructed tracks, corresponding to 18 swarms of approximately ten mosquitoes.

2- The discussion section appears too general and superficial. For instance, the last sentence on vector control should be removed or expanded. In principle any study on mosquitoes may be considered useful for vector control. The authors need to be specific on how their findings could be useful for applications in vector control. Another example in this direction is the paragraph at line 10 pag.7 (second last paragraph) of the discussion section, about male-female and male-male interaction. This paragraph does not add anything to the overall message of this work, and should be removed or revised.

3- Authors do not address potential effects of the laboratory environment on mosquito behavior.

4- Rules defining the models should be at least mentioned in the main text. Otherwise the reader is forced to go to SI.

5- The comparison between model and data shows that simulations produce saccades symmetric on the plane xy and yz, against a clear asymmetry in the data. The authors should comment on this discrepancy. The same symmetry/asymmetry is present in the comparison of the straight flight.

Reviewer #3: This work describes the swarming of malaria mosquitoes based on individual 3D tracks in the lab and investigates the underlying behaviour rules of their swarms using an agent-based model. I find the paper clear and concise, and a very nice fit to the journal. It is well-written and with nice visualization of results. I have however a few remarks and questions that I think should be addressed before the paper’s publication, and some comments that I think can increase the paper’s contribution and impact. Please find them below:

- Why there are no methods in the main text? Some experimental conditions should be mentioned in the results text, especially how many mosquitoes were tracked and for what duration. Same for some main aspects of the simulations.

- I understand this paper is an extension of the Poda et al. 2024 work. Given the overlap, I think that the authors should more clearly state (perhaps even in the introduction/results) and discuss in the end the differences/extensions in empirical (especially for sections ‘Flight kinematics of individual swarming mosquitoes’ and ‘Variations in flight kinematics within the swarm volume) and modelling findings (since a different model was used in the previous study).

- Fig. 1A is exactly the same as in ref [37], Poda et al. 2024, without a reference in the caption. Not sure what’s the journal’s policy is on this.

- The comparison with other species could be made a bit more specific, e.g., pg.7 line 5, what different environmental cues? Are there any relevant findings from other swarming insects?

- I find the number 4.5% reduction of the viewing angle as the trigger to a turning manoeuvre a bit too specific, perhaps the authors could elaborate more in their Discussion about it.

- Model description: As I mentioned already above, some more details about the simulations should be added in the main text. A table with all the parameters and their default values should also be added in the Supplementary. Additionally, despite the model description being clear, I would recommend that the authors could at least use more terminology from the ODD protocols (to not write a full one) [1]. This will support both the reproducibility of the agent-based model as well as strengthen its link to the literature.

- Pg 7 line 19: ‘mosquito swarms as an ideal system for studying animal collective behavior’ I found this opposing the conclusion that mosquito swarms are ‘inherently different from flocking and schooling’. Perhaps the authors meant collective behaviour of insect swarms? Saying that, a paragraph on comparisons with findings in other insect swarms would be nice addition to the Discussion.

- Maximum swarm size from Fig S7 seems to be 26, is this the maximum over the experiments or just the one used for the plot? In general, are the swarm sizes here reflecting the ones found in the field? Perhaps the authors could expand a bit on their discussion pg 6 line 44.

- Fig. S4 f and h: there seem to be larger differences between empirical data and simulations here, perhaps the authors can provide some clarification of why this is the case in the caption or text.

- Pg. 6, line 39: ‘previously suggested apparent long distance interactions’ I think that this statement needs references.

Minor comments:

- pg. 3 line 40: unnecessary second ‘and’ ?

- pg. 4 line 19: ‘remain’

- Ref 37: bioRxiv references instead of the published version

- title: ‘emerge’ rather than ‘emerges’ if it refers to the dynamics and not the swarming.

References:

[1] Grimm, Volker, Steven F. Railsback, Christian E. Vincenot, Uta Berger, Cara Gallagher, Donald L. DeAngelis, Bruce Edmonds et al. "The ODD protocol for describing agent-based and other simulation models: A second update to improve clarity, replication, and structural realism." Journal of Artificial Societies and Social Simulation 23, no. 2 (2020).

**********

**Have the authors made all data and (if applicable) computational code underlying the findings in their manuscript fully available?**

The PLOS Data policy requires authors to make all data and code underlying the findings described in their manuscript fully available without restriction, with rare exception (please refer to the Data Availability Statement in the manuscript PDF file). The data and code should be provided as part of the manuscript or its supporting information, or deposited to a public repository. For example, in addition to summary statistics, the data points behind means, medians and variance measures should be available. If there are restrictions on publicly sharing data or code —e.g. participant privacy or use of data from a third party—those must be specified.

Reviewer #1: **No:** Unless I have missed, the code should be available via Code S1 and S1 was not provided.

Reviewer #2: Yes

Reviewer #3: Yes

**********

PLOS authors have the option to publish the peer review history of their article (what does this mean?). If published, this will include your full peer review and any attached files.

If you choose “no”, your identity will remain anonymous but your review may still be made public.

**Do you want your identity to be public for this peer review?**  For information about this choice, including consent withdrawal, please see our Privacy Policy.

Reviewer #1: No

Reviewer #2: No

Reviewer #3: No

[NOTE: If reviewer comments were submitted as an attachment file, they will be attached to this email and accessible via the submission site. Please log into your account, locate the manuscript record, and check for the action link "View Attachments". If this link does not appear, there are no attachment files.]

**Figure resubmission:**

While revising your submission, we strongly recommend that you use PLOS’s NAAS tool (https://ngplosjournals.pagemajik.ai/artanalysis) to test your figure files. NAAS can convert your figure files to the TIFF file type and meet basic requirements (such as print size, resolution), or provide you with a report on issues that do not meet our requirements and that NAAS cannot fix.

After uploading your figures to PLOS’s NAAS tool - https://ngplosjournals.pagemajik.ai/artanalysis, NAAS will process the files provided and display the results in the "Uploaded Files" section of the page as the processing is complete. If the uploaded figures meet our requirements (or NAAS is able to fix the files to meet our requirements), the figure will be marked as "fixed" above. If NAAS is unable to fix the files, a red "failed" label will appear above. When NAAS has confirmed that the figure files meet our requirements, please download the file via the download option, and include these NAAS processed figure files when submitting your revised manuscript.

**Reproducibility:**

?>

Attachment Submitted filename: Review.pdf

# Author comment: Author response to Decision Letter 1

**DOI:** 10.1371/journal.pcbi.1014685.r002

21 Nov 2025

Attachment Submitted filename: Response to reviewers.pdf

# Peer review: Decision Letter 1

**DOI:** 10.1371/journal.pcbi.1014685.r003

**Authors:** Tobias Bollenbach

19 Dec 2025

PCOMPBIOL-D-25-00797R1

The complex swarming dynamics of malaria mosquitoes emerge from simple minimally-interactive behavioral rules

PLOS Computational Biology

Dear Dr. Cribellier,

Thank you for submitting your manuscript to PLOS Computational Biology. As with all papers, your manuscript was reviewed by members of the editorial board. Based on our assessment, we have decided that the work does not meet our criteria for publication and will therefore be rejected. If external reviews were secured, reviewers' comments will be included at the bottom of this email.

We are sorry that we cannot be more positive on this occasion. We very much appreciate your wish to present your work in one of PLOS's Open Access publications. Thank you for your support, and we hope that you will consider PLOS Computational Biology for other submissions in the future.

Yours sincerely,

Tobias Bollenbach

Section Editor

PLOS Computational Biology

**Additional Editor Comments (if provided):**

While two of the reviewers are now mostly satisfied with the revisions, reviewer #2's major concerns remain largely unresolved. Here, we tend to side with reviewer #2. In particular, we agree that:

- Artifacts caused by the experimental setup (e.g., cage geometry) cannot be ruled out based on the presented data. Proper controls for this would be essential. Ultimately, the conclusions based on the current data are too strong.

- The dynamic range of swarm sizes in the experiments is insufficient to support the current conclusions.

Resolving these issues will require either additional experimental evidence or drastic changes to the main conclusions (see the specific suggestions of reviewer #2). The latter option would require changes that go beyond what can be done in a major revision and would fundamentally alter the manuscript.

If you believe you can address these concerns, we are willing to consider a substantially revised manuscript as a new submission.

[Note: HTML markup is below. Please do not edit.]

**Reviewers' Comments (if peer reviewed):**

Reviewer's Responses to Questions

**Comments to the Authors:**

**Please note here if the review is uploaded as an attachment.**

Reviewer #1: I thank the authors for substantially improving the manuscript. The revisions clarify the conceptual framework and highlight the manuscript’s contributions to the literature much better.

I find the addition of individual-trajectory comparisons highly useful in demonstrating that the swarming behavior is scale-free. While I do not entirely agree that the absence of group-size effects conclusively indicates little or no contribution of social cues, the authors are entitled to argue for parsimony (i.e., that environmental cues alone drive this behavior) given the breadth of analysis presented.

Echoing another reviewer’s comments, I believe further work is needed to elucidate the precise relationship between environmental factors and swarming dynamics. I therefore look forward to the authors’ future contributions on this topic.

Reviewer #2: Without additional experiments, the conclusions of this work, particularly the claim of mosquito alignment with the sunset direction, are not sufficiently supported by the data. Therefore, I do not recommend publication of this work in its current version. Below, I provide comments on the authors’ responses. To clearly distinguish my remarks from those of the authors, each paragraph is prefaced with “Reviewer” or “Authors”, respectively.

Authors: Reply 2.2: We thank the reviewer for raising this important point. We agree that the observed flight direction and saccadic maneuvers could, in principle, be influenced by the physical constraints of the flight arena. To address this, we performed a dedicated analysis quantifying the proximity of mosquitoes to the arena walls during saccadic turns (see new Fig. S11).

Reviewer: The distance from the wall at which saccades occur is indeed an interesting quantity; however, the analysis presented here cannot objectively exclude the possibility that the cage interferes with mosquito flight dynamics.

In Figure S11, authors show the distributions of these distances along the two axes x and y, and in the discussion they focus on mean values. I see two weaknesses in this approach. First, the most informative part of these two distributions is not the mean but the left tail, which measures the shortest distances from the wall. Second, as discussed in detail below, the comparison with the distance of collision avoidance is not motivated to assess whether there is an influence of walls on mosquitoes dynamics.

The two histograms shown in Fig.S11 show that the distributions are quite different between the two axes. Along the x-axis the left tail indicates that mosquitoes do not get closer than 10/15cm to the wall. Along the y-axis, instead, saccades are found at very short distances from the walls, where it is unlikely that the effects of confinement are negligible. A substantial fraction of saccades is also found in the region (highlighted in red) that defines the distance of the swarm from the swarming arena. These histograms would be conclusive only if the left tails were comparable along both axes, indicating similar wall-related behavior in the x and y directions.

We cannot tell from this data, whether this is a genuine effect of the alignment with the light, or instead it is the effect of the confinement. The only way to discriminate between these two potential interpretations is to perform experiments changing the position of the light source and checking swarming behavior under these different conditions.

Authors: Our results show that mosquitoes turned at average distances of 45 ± 8.5 cm from the side walls and 23 ± 7.1 cm from the front/back walls—equivalent to 75 and 38 body lengths, respectively. These distances are an order of magnitude greater than the inter-individual distances that trigger collision avoidance (~2.5 body lengths; Fig. 5K, [8]).

Reviewer: The comparison between the distance from the walls at which saccades occur and the distance that triggers collision avoidance with other insects is not motivated. Such a comparison seems to imply that mosquitoes avoid flying toward a wall using the same mechanism involved in collision avoidance with other insects, which is unlikely. A wall imposes a hard constraint on the mosquitoes’ flight phase space, whereas the presence of another nearby insect does not. This argument would have been compelling if the authors could objectively prove that the presence of a wall would influence mosquitoes dynamics in the same way as another insect flying nearby, which is not shown here. Otherwise, the comparison remains quantitative but strongly speculative.

Authors: Additionally, the preferred flight direction—orthogonal to the sunset horizon—aligns with the short axis of the arena (y-axis), which is counterintuitive if wall effects were dominant. If cage geometry were driving the behavior, we would expect alignment along the long axis (x-axis), which is not observed.

Reviewer: This argument is too speculative and not supported by the data. Intuitiveness or counterintuitiveness cannot be used to assess the validity of an interpretation, particularly in biology. Therefore, I invite the authors to either revise or remove this part of the manuscript, or to support this conjecture with appropriate references from the literature.

Authors: Together, these findings strongly suggest that wall avoidance does not explain the observed saccadic maneuvers or flight orientation. Instead, the data support our interpretation that these behaviors are visually mediated interactions with the marker.

We acknowledge the reviewer’s suggestion for further experiments to disentangle these effects. While such tests (e.g., varying sunset direction or marker shape) would be valuable for refining our understanding of swarm geometry, they fall outside the main scope of this study. Our primary aim is to identify the behavioral rules underlying mosquito swarming, rather than to characterize the exact shape or orientation of the swarm. Moreover, our newly-added analysis based on current data already provide strong evidence that the observed behaviors are not artifacts of the experimental setup. We have added this new analysis and expanded the discussion accordingly (lines 420-426 and 564-570; Fig. S11).

Reviewer: I do not think that further experiments will be relevant to refining the understanding of swarm geometry. They are necessary to confirm that the preferred direction is related to the light, which is one of the behavioral rules highlighted in the manuscript, and to actually prove that the same behavior is observed under a different setup. If this is not the case, i.e, if authors believe that the fact that the preferred direction of flight is relevant as a result of their study but only saccades are relevant, they should revise the manuscript and remove the claims about the direction of light from the abstract as well as from the result section, leaving it at a pure speculative level in the discussion.

Authors: Reply 2.3: We appreciate the reviewer’s insightful comment. Indeed, distinguishing between attraction to conspecifics and attraction to an environmental marker is challenging in collective behavior studies.

Two key findings support that the marker is the primary swarm attractor:

1. Stable swarm position: Swarms consistently remained above the marker for extended periods. If attraction were primarily to conspecifics, we would expect positional drift over time—especially given that our chamber design allows for such movement. This drift was never observed.

2. Swarming by single individuals: We recorded flight kinematics from the onset of swarming (single mosquito) to fully developed swarms (up to 26 individuals; Fig. S8). Even solitary males exhibited sustained swarming above the marker, with kinematics comparable to those in larger swarms (see Reply 1.5 for details). This suggests that attraction to the marker is independent of swarm size. Based on these results, attraction to the marker remains the most parsimonious explanation for swarm cohesion. To test this, we developed an agent-based model using the marker as the sole attractor, with collision avoidance as the only conspecific interaction. This minimal model successfully reproduced swarm kinematics across group sizes, indicating that explicit attraction between individuals is not required.

While we cannot entirely exclude conspecific attraction, our data show it is not necessary to explain mosquito swarming. We have updated the manuscript to include these analyses and expanded the discussion on the model’s assumptions and limitations (lines 429-437 and 537-549)

Reviewer: I agree that the attraction to the marker is the most parsimonious choice. However, I would not use the argument of a single mosquito flying on the marker to assess its validity. The crucial role of environmental markers in insect swarms is already well established in the literature, and inter-individual interactions should be viewed not as an alternative to marker attraction, but as an additional ingredient. The absence of positional drift, or the ability of a single individual to remain above the marker, does not discriminate between an underlying interaction driving swarming behavior and a response to external cues; by this logic, the present work, as well as much of the existing swarming literature, would not be needed.

Despite this remark, if the authors wanted only to show that stable swarms can be reproduced with very simple rules, I think the manuscript would benefit from a clearer comparison with existing models. For example, models developed in Ouellette’s lab are able to reproduce stable swarms without explicitly incorporating inter-individual interactions. In this context, it is not clear in what sense the present model represents an advance over the current state of the art.

Authors: Reply 2.4: As requested by the reviewer, we now clarify in detail the number of swarming experiments performed (six swarms with three recordings per swarm), and the number of swarming mosquitoes per swarm (from 1 to 26 individuals) thorough the manuscript. Moreover, we now explicitly state the sample sizes used for each figure in its figure legend. We also discussed how the number of swarming mosquitoes did not seem to affect their swarming behavior (lines 537-549).

Reviewer: I thank the authors for adding details on the number of swarming experiments. However, I would use much more caution, all over the manuscript, in claiming that the swarm size does not seem to affect swarming behavior, since the data presented here refer to a quite limited size (from 1 to 26 mosquitoes), in particular when compared to field swarms, which may be composed of thousands of individuals, as shown in [1]. In this regard, the section “Variations in flight kinematics with swarm size” (lines 419-437) should be revised or removed.

[1] Ouédraogo TFX, Sawadogo SP, Millogo AA, Niang A, Ouedraogo J, Ouattara SB, Cribellier A, Namountougou M, Dabiré RK, Muijres FT, Diabaté A. Characterization of factors influencing swarm dynamics and mating efficiency in Anopheles coluzzii. Parasit Vectors. 2025 Nov 27. doi: 10.1186/s13071-025-07151-w. Epub ahead of print. PMID: 41310880.

Authors: Reply 2.5: As requested by the reviewer, we extended the discussion section in the revised manuscript. Hereby, we including a comparison of the studied mosquito swarming with other group behaviors (lines 599-612), we revising the paragraph discussing how our study informs us on male-female interactions in mosquito mating swarms (lines 590-598), and we expended the part of the discussion about vector control (lines 613-625). We hope that the reviewer is satisfied with the extended discussion section.

Reviewer: In general, I am not particularly in favor of general discussions that are not directly related to the results. I appreciate the authors' effort to broaden the discussion of the relevance of their work, but I do not consider it necessary, nor do I think it adds anything substantial to the main message of the manuscript. The relevance of this study seems primarily related to fundamental biology, which is as valuable as applied biology, and I would prefer a discussion oriented at fundamental biology.

Authors: Reply 2.6: As mentioned in our Reply 2.2, we have added a dedicated analysis addressing potential effects of the laboratory environments on the recorded mosquito behaviors. We discuss this in the methods, results and discussion sections of the revised manuscript (lines 420-426 and 561-570; see also Fig. S11).

Reviewer: As already discussed above, I do not think that this analysis is robust enough and does not prove that mosquitoes behavior is not affected by the experimental setup.

Authors: Reply 2.5: We have now included the material and methods section in the main text, thus resolving this issue.

Reviewer: I thank the authors for this change.

Authors: Reply 2.6: We believe that the reviewer refers to results presented in Fig. S4. In that figure, we compare the 2D heat maps of mean angular speed or of saccade-to-straight flight ratio in the XY and YZ plans. When comparing the heatmaps from the experimental data and from simulated data, we see that both real and simulated mosquitoes have on average higher angular speeds on the boundaries of the swarm, thus showing high proportion of saccadic maneuvers at the edges, and high proportion of straight flights in the middle of the swarm. These 2D distributions of both experimental and simulated data follow a clear axis symmetry around the central vertical axis of the swarm (Fig. S4). Therefore, it is unclear to us which asymmetry the reviewer refers to.

There are, however, key differences between the distributions of the experimental data and simulated data. Most importantly, in the simulations we observe high angular speed and high proportion of saccades around the swarm boundaries (Fig. S4c,d and Fi. S4g,h). This is due to a simplification of our agent-based model where mosquitoes will only trigger a saccade if they are going to exactly cross the swarm boundaries (i.e. binary threshold), while in reality they can trigger a saccade a bit before or after crossing the boundary. This results for the experimental swarms in more diffused distributions of saccades in space (Fig. S4c,d and Fi. S4g,h) and less defined swarm boundaries (as visible on Fig. 5C,D). We have now included a comparison of the experimental and simulation results in the discussion (lines 520-536 and 571-578), highlighting these similarities and differences

Reviewer: I apologize for not having specified that I was referring to Figure S4, as correctly guessed by the authors. In Figure S4 (panels b and d), the authors show the distributions on the xy-plane of the angular speed and of the saccade-to-straight flight ratio. In both plots, I see an asymmetry between x and y directions, which does not seem to be in agreement with the symmetry around the central vertical axis claimed by the authors.

As stated by the authors, both real and simulated mosquitoes have on average higher angular speeds on the boundaries of the swarm, thus showing a high proportion of saccadic maneuvers at the edges, and high proportion of straight flights in the middle of the swarm. However, the experimental distributions also show a clear axis dependence: at the edges of the swarm along the y-direction, mosquitoes reach higher angular speed than those at the edges of the swarm along the x-direction. Similarly, the saccade-to-straight flight ratio measured at the swarm boundaries exhibits a directional dependence.

This asymmetry is not present in the simulations. This discrepancy is not discussed in the manuscript, and I do not think it may be due to the binary threshold for the boundaries used in the simulations.

Reviewer #3: The authors did a great job addressing my comments. The updated methods are indeed much clearer and the model description enables model reproducibility. The data and code repository is also well structured and easily accessible. I am happy to recommend this article for publication but please find some minor comments on 3 points below:

- I like the additional analysis and text regarding swarm size effects. I was wondering whether density effect can start playing a role in larger aggregations, where there may be more competition for instance for positions closer to the center of the attractor and females. The authors nicely discuss this in lines 537-549 that usually the swarms don’t reach higher densities in nature since the volume of the swarm usually also increases, but if there is any additional ecological insight in the literature (on what the males compete for or on female preference [eg. 1]) could be a nice addition here to solidify the argument (or a mention that this gap of research could be addressed with more data in the future). For instance, under the prism of collective behavior [2], why would interactions among males not matter on their mating success?

Minor:

Lines 436 (‘confirms previous suggestions that a mosquito mating swarm of a single individual can exist [21, 48]’) and 541( ‘supports the observation that even a single mosquito can display swarming behavior in the absence of conspecifics [21,48]’ ) are rather overlapping, I think the first one in the results is unnecessary and can be omitted (also because a single individual shouldn’t be called a swarm, but rather showing swarming behavior as the authors write later).

[1] Ng'habi, K. R., Huho, B. J., Nkwengulila, G., Killeen, G. F., Knols, B. G., & Ferguson, H. M. (2008). Sexual selection in mosquito swarms: may the best man lose?. Animal Behaviour, 76(1), 105-112. https://doi.org/10.1016/j.anbehav.2008.01.01

[2] Rathore, A., Isvaran, K., & Guttal, V. (2023). Lekking as collective behaviour. Philosophical Transactions of the Royal Society B, 378(1874), 20220066. https://doi.org/10.1098/rstb.2022.0066

- Comparisons with other species: I find that the new discussion paragraph nicely puts the results in a wider context (lines 599-612). I only find unclear what the authors mean by framework (line 611) here (a collection of metrics? Or the behavioral algorithm of the model?). Perhaps they could add a clarification. A nice link here could also be with a recently developed framework to compare collective motion across species (‘swarm verse’ [2]) that has been focused only on vertebrates. Mating swarms of insects seems like an ideal space to adjust this framework to, expanding towards invertebrates.

[3] Papadopoulou, Marina, Simon Garnier, and Andrew J. King. "swaRmverse: An R package for the comparative analysis of collective motion." Methods in Ecology and Evolution 16.1 (2025): 29-39. https://doi.org/10.1111/2041-210X.14460

- In the author summary, the sentence: ‘how male malaria mosquitoes form mating swarms’ may make readers think about long range attraction to environmental cues and one another to ‘initiate’ a swarm rather than how the swarming characteristics arise. Perhaps the authors should revise/clarify this.

**********

**Have the authors made all data and (if applicable) computational code underlying the findings in their manuscript fully available?**

The PLOS Data policy requires authors to make all data and code underlying the findings described in their manuscript fully available without restriction, with rare exception (please refer to the Data Availability Statement in the manuscript PDF file). The data and code should be provided as part of the manuscript or its supporting information, or deposited to a public repository. For example, in addition to summary statistics, the data points behind means, medians and variance measures should be available. If there are restrictions on publicly sharing data or code —e.g. participant privacy or use of data from a third party—those must be specified.

Reviewer #1: Yes

Reviewer #2: Yes

Reviewer #3: Yes

**********

PLOS authors have the option to publish the peer review history of their article (what does this mean?). If published, this will include your full peer review and any attached files.

If you choose “no”, your identity will remain anonymous but your review may still be made public.

**Do you want your identity to be public for this peer review?**  For information about this choice, including consent withdrawal, please see our Privacy Policy.

Reviewer #1: No

Reviewer #2: No

Reviewer #3: No

[NOTE: If reviewer comments were submitted as an attachment file, they will be attached to this email and accessible via the submission site. Please log into your account, locate the manuscript record, and check for the action link "View Attachments". If this link does not appear, there are no attachment files.]

# Author comment: Author response to Decision Letter 2

**DOI:** 10.1371/journal.pcbi.1014685.r004

18 Mar 2026

Attachment Submitted filename: Response to reviewers 2.pdf

# Peer review: Decision Letter 2

**DOI:** 10.1371/journal.pcbi.1014685.r005

**Authors:** Tobias Bollenbach

7 May 2026

The complex swarming dynamics of malaria mosquitoes emerge from simple minimally-interactive behavioral rules

PLOS Computational Biology

Dear Dr. Cribellier,

Thank you for submitting your manuscript to PLOS Computational Biology. After careful consideration, we feel that it has merit but does not fully meet PLOS Computational Biology's publication criteria as it currently stands. Therefore, we invite you to submit a revised version of the manuscript that addresses the points raised during the review process.

Please submit your revised manuscript by Jul 07 2026 11:59PM. If you will need more time than this to complete your revisions, please reply to this message or contact the journal office at ploscompbiol@plos.org. When you're ready to submit your revision, log on to https://www.editorialmanager.com/pcompbiol/ and select the 'Submissions Needing Revision' folder to locate your manuscript file.

Please include the following items when submitting your revised manuscript:

* A letter that responds to each point raised by the editor and reviewer(s). You should upload this letter as a separate file labeled 'Response to Reviewers'. This file does not need to include responses to formatting updates and technical items listed in the 'Journal Requirements' section below.

* A marked-up copy of your manuscript that highlights changes made to the original version. You should upload this as a separate file labeled 'Revised Manuscript with Track Changes'.

* An unmarked version of your revised paper without tracked changes. You should upload this as a separate file labeled 'Manuscript'.

We look forward to receiving your revised manuscript.

Kind regards,

Tobias Bollenbach

Section Editor

PLOS Computational Biology

**Additional Editor Comments:**

The reviewers have raised several issues that still need to be addressed. In particular, please address Reviewer #2's concerns about the compatibility of the behavioral rules with the new dataset. Additionally, ensure that the conclusions are not overstated, particularly in the abstract and summary sections of the main text.

**Journal Requirements:**

1) Please ensure that the funders and grant numbers match between the Financial Disclosure field and the Funding Information tab in your submission form. Note that the funders must be provided in the same order in both places as well. Currently, "Sectorplan Biology of the Dutch Ministry of Education, Culture and Science (OCW)" is missing from the Financial Disclosure field.

Note: If the reviewer comments include a recommendation to cite specific previously published works, please review and evaluate these publications to determine whether they are relevant and should be cited. There is no requirement to cite these works unless the editor has indicated otherwise.

**Reviewers' comments:**

Reviewer's Responses to Questions

**Comments to the Authors:**

**Please note that one review is uploaded as an attachment.**

Reviewer #1: .

Reviewer #2: I really appreciate the effort made by the authors to expand the analyzed data and extend the discussion. However, I have some concerns regarding the compatibility of the Feugere male dataset with the model introduced in the present work. At this stage, I am unable to recommend publication of the manuscript.

For a model to be meaningful, it must demonstrate predictive power on datasets not used to fit the parameters. Given the availability of multiple datasets, the authors could have followed a standard cross-validation procedure: using the Poda dataset to fit the model parameters, as they do, and then quantitatively assessing the extent to which the Faugere datasets reproduce the predicted behavior. This step is, in my view, not properly addressed and it lacks a quantitative analysis.

More specifically, my concerns regarding the compatibility of the model with the Feugere male dataset involve both Behavioral rule 1 and Behavioral rule 2, as detailed below.

Behavioral rule 1: Swarming mosquitoes preferentially fly straight through the swarm center, where the marker viewing angle is maximal. They then continue flying straight, until the marker viewing angle falls below a threshold value, which triggers a turning maneuver.

Behavioral rule 1 implies that the swarm center corresponds to the marker center, and that there is symmetry in the structure of the swarm with respect to the marker position. Although the Feugere male dataset exhibits a structural symmetry around its own vertical axis, it is shifted in the y-axis towards the negative direction, see Fig.S12a-b. As a result, part of the swarm is consistently out of the marker, and mosquitoes do not appear to show a preference for the region where the viewing angle is maximized, namely the center of the marker. This discrepancy suggests that behavioral rule 1 fails to predict and reproduce the position of the Feugere male dataset, and therefore highlights the lack of generality of the model. Maximizing the viewing angle of the marker may therefore not be the mechanism underlying swarm formation and spatial structure.

Behavioral rule 2. The turning maneuver consists of a rapid saccadic turn within the horizontal plane, with biases towards the swarm center and along the sunset axis (towards and away from the sunset position) [32]. After the turn, the mosquito continues to fly in a straight line (rule 1).

In my opinion this is the most critical aspect. There is a lack of quantitative assessment of the existence of a preferred direction of flight, and authors rely only on a qualitative evaluation of the plots shown in Fig.S13g and Fig.S15f. In detail, my concerns are the following:

1- In Fig.15f, I do not see clear evidence of a preferred flight direction. The distribution is indeed radially symmetric, in contrast with Fig.S15h (female dataset) and Fig.S4 (Poda dataset)

2- I am struggling to understand how distributions in Fig.S13g are normalized. By definition, the integral of each distribution should be equal to 1. But the distributions in this panel seem to cover areas that are not comparable with each other. To be more clear, let’s focus on the dark red and dark blue distributions. The dark red has a well pronounced peak at -90deg and it is quite narrow (the radii of the distributions are very small except for a very narrow portion close to -90deg). The dark blue is instead much broader, covering the entire [-180deg:180deg] interval with a peak at -90deg. By definition of probability distribution, its peak at -90deg cannot have the same radius of the dark red, as instead it has in Fig.S13g: if both distributions are properly normalized, the broader distribution should exhibit a lower peak. This makes the area of the dark blue much larger than the area of the dark red, giving the misleading impression that the two peaks (the one of the dark red and the one of the dark blue) have similar statistical significance. Authors are probably scaling one of the two distributions.

3- The distributions in Fig.S13g for the Feugere male dataset are computed separately for y>0 and y<0, as done for all the other datasets. This choice would have been reasonable if the swarm were centered with the marker (see comment above). But in this specific situation y=0 does not correspond to any physically meaningful reference position. The distributions should instead be defined with respect to the actual swarm center. Choosing y=0 as a splitting point, likely causes the presence of a significant portion of the dark blue distribution in the region of positive angles, as well as the imbalance between the dark and light blue distributions. Also note that, because of the color code and of the transparency of the plot, the portion of the dark blue distribution in the positive angles region is barely visible, unless when zooming in the figure. Its visibility could be improved using different colors.

4- To better assess the presence of a preferred flight direction, it would be useful to include, for the Feugere male dataset, a figure showing the distribution of the azimuth angle between two saccades as a function of mosquito positions, analogous to Fig.3c-d and Fig.S5a-b for the Poda dataset.

5- It would also be important to introduce a quantitative metric to characterize the degree of alignment in the flight directions, rather than relying solely on qualitative interpretation of the distributions.

Reviewer #3: I think the authors did a great job addressing my previous comments, I do not have any follow up. In relation to the larger discussion with the comments of Reviewer 2, I wanted to mention the following points:

1) I think the newly analysed published dataset is an excellent addition to the paper that indeed provides further support to the findings.

2) Comment 2.2: I agree with the authors’ arguments and I find the extended discussion clear and effective in communicating the response to the concerns of Reviewer 2.

2) Comment 2.3: Inter-individual distances are often used as a unit metric, so I don’t find it peculiar or confusing in the manuscript. I see that it may be misleading for an interdisciplinary audience and thus the clarification the authors added is a good improvement.

3) Comment 2.6: I agree with Reviewer 2 in relation to the markers and inter-individual attraction. I think a stronger clarification on the comparison with existing models would be beneficial, and I think the authors did a good job explaining this in their new discussion. As the authors mention, testing minimal individual-level rules necessary to reproduce collective patterns is an invaluable insight from such agent-based models with important theoretical contributions despite the possibility of more complex behaviours of individual organisms.

4) Concerning the wall effects overall, as the authors note at Reply 2.9, experimental setups may always have an effect on behaviour and usually cannot be ruled out, it is the cost of having a more standardised alternative to a study in the field. For instance, wall effects are present in most studies with laboratory experiments in fish schools. I think the authors did the best they could in testing these effects, and the new dataset further supports that their conclusions are not overstated. In my opinion, the new limitations section of the Discussion covers everything quite extensively.

Overall, I think that more cautious interpretation is always a plus, especially when studying a complex multi-agent system (where causality is very hard to prove empirically) and when simulations are used, given other potential underlying rules that may be able to reproduce the same emergent properties. Thus, I find the revised manuscript highly improved and the claims of the authors now well balanced and justified. I am happy to recommend its publication.

Minor remarks:

- Lines 560, I think there is a typo in the first new sentence. Perhaps a reference is also needed in the first sentence of that paragraph.

- There is some repetition in the new part of the discussion, eg. lines 645 vs lines 655. Perhaps the first short paragraph is not necessary and can be merged with the following 2 for conciseness.

**********

**Have the authors made all data and (if applicable) computational code underlying the findings in their manuscript fully available?**

The PLOS Data policy requires authors to make all data and code underlying the findings described in their manuscript fully available without restriction, with rare exception (please refer to the Data Availability Statement in the manuscript PDF file). The data and code should be provided as part of the manuscript or its supporting information, or deposited to a public repository. For example, in addition to summary statistics, the data points behind means, medians and variance measures should be available. If there are restrictions on publicly sharing data or code —e.g. participant privacy or use of data from a third party—those must be specified.

Reviewer #1: Yes

Reviewer #2: Yes

Reviewer #3: Yes

**********

PLOS authors have the option to publish the peer review history of their article (what does this mean?). If published, this will include your full peer review and any attached files.

If you choose “no”, your identity will remain anonymous but your review may still be made public.

**Do you want your identity to be public for this peer review?**  For information about this choice, including consent withdrawal, please see our Privacy Policy.

Reviewer #1: No

Reviewer #2: No

Reviewer #3: No

[NOTE: If reviewer comments were submitted as an attachment file, they will be attached to this email and accessible via the submission site. Please log into your account, locate the manuscript record, and check for the action link "View Attachments". If this link does not appear, there are no attachment files.]

**Figure resubmission:**

**Reproducibility:**

?>

Attachment Submitted filename: Comments.pdf

# Author comment: Author response to Decision Letter 3

**DOI:** 10.1371/journal.pcbi.1014685.r006

1 Jun 2026

Attachment Submitted filename: Response to reviewers 3.pdf

# Peer review: Decision Letter 3

**DOI:** 10.1371/journal.pcbi.1014685.r007

**Authors:** Tobias Bollenbach

4 Aug 2026

Dear Dr. Cribellier,

We are pleased to inform you that your manuscript 'The complex swarming dynamics of malaria mosquitoes emerge from simple minimally-interactive behavioral rules' has been provisionally accepted for publication in PLOS Computational Biology.

Before your manuscript can be formally accepted you will need to complete some formatting changes, which you will receive in a follow up email. A member of our team will be in touch with a set of requests.

Please note that your manuscript will not be scheduled for publication until you have made the required changes, so a swift response is appreciated.

IMPORTANT: The editorial review process is now complete. PLOS will only permit corrections to spelling, formatting or significant scientific errors from this point onwards. Requests for major changes, or any which affect the scientific understanding of your work, will cause delays to the publication date of your manuscript.

Should you, your institution's press office or the journal office choose to press release your paper, you will automatically be opted out of early publication. We ask that you notify us now if you or your institution is planning to press release the article. All press must be co-ordinated with PLOS.

Thank you again for supporting Open Access publishing; we are looking forward to publishing your work in PLOS Computational Biology.

Best regards,

Tobias Bollenbach

Section Editor

PLOS Computational Biology

***********************************************************

Reviewer's Responses to Questions

**Comments to the Authors:**

**Please note here if the review is uploaded as an attachment.**

Reviewer #1: I am thoroughly satisfied with the current state of the paper and can only recommend it for publication.

Reviewer #2: This manuscript is now at its fourth round of revision. Despite the new figure and the introduction of the Rayleigh test, I find that the data interpretation is still flawed and that there is an overall lack of scientific rigor. Therefore I cannot recommend this work for publication. My suggestion to the authors is to focus their analysis on the saccades, which represent a robust feature across the datasets, instead of attempting to define a specific behavioral model.

Authors present data from three datasets: the original Poda dataset on males, and two datasets (one on males, one on females) from Feugere. They define a behavioral model, designed and fitted on the Poda dataset, based on three simple behavioral rules. My main concerns are about the incompatibility of the male Feugere data with the model and the authors’ justifications for this incompatibility, which in my opinion compromise the validity of the entire work.

Behavioral rule 1 (BR1) concerns swarm structure, specifically involving mosquitoes’ viewing angle of the marker. The authors do not provide a quantitative measure to test whether, and to what extent, the Feugere datasets follow this rule. However, based on the figures they provide, the female swarm appears almost centered over the marker, hence in principle compatible with BR1. In contrast, the male swarm is shifted along the y direction, thereby showing a lack of compatibility with BR1. If the viewing angle were the dominant factor shaping the structure of the swarm, mosquitoes above the center of the marker would be in the ideal condition, with maximum viewing angle; therefore they should not exhibit a tendency to turn as they do, and the swarm should be centered on the marker. The authors justify this incompatibility in the male data by pointing to the different geometry of the marker (square and flat for Poda, elevated, cylindrical and asymmetric for Feugere). However, this justification is directly contradicted by the female data. The female behavior demonstrates that the Feugere setup does not physically or visually preclude the emergence of BR1-like swarm structures. Therefore, the failure of the male dataset cannot be attributed to the experimental geometry alone, but rather indicates that the model's behavioral assumptions lack the necessary robustness to generalize across datasets.

Behavioral rule 2 (BR2) concerns the direction of flight related to saccadic turns, which is biased towards the sunset direction. Through circular probability distribution and the Rayleigh test, the authors evaluate at both a qualitative and quantitative level whether the Feugere data are compatible with BR2. The female distribution is quite similar to that of the Poda dataset, with two large peaks corresponding to the direction of the sunset and a pronounced minimum in the orthogonal direction. In contrast, after correcting the severe methodological error of scaling the plot, the male distribution is only slightly skewed in the direction of the sunset and lacks a pronounced minimum, indicating that all directions are almost equivalent. This visual observation is confirmed by the Rayleigh test, which yields r=0.371 for females and r=0.138 for males, expressing a moderate and weak alignment respectively. Compared with the r=0.426 of the Poda dataset, these values suggest that the female data are compatible with both Poda and BR2, while the male data are not.

In this regard, the manuscript presents three major issues.

First, depending on the context, the authors refer either to p-values or to r to assess the significance of the alignment they found. But these two parameters are inherently different. The p-value is a measure of the statistical significance of the test, and it is heavily dependent on the sample size; a low p-value can be completely driven by a large number of samples, even in the case of a negligible alignment effect. The actual measure of the alignment strength is given by r. The authors ultimately contradict themselves in one single sentence in the caption of Fig.S12, where they write “Both males and females horizontal flight directions show significant alignment with sunset axis (Rayleigh test on axial data, (p < 0.001, r = 0.138 and 0.371, respectively showing weak and moderate alignment)”. Moreover, going from r = 0.426 (Poda) to r = 0.138 (male Feugere data) we move from a highly structured alignment to near-total uniformity. Claiming that an r = 0.138 validates the model, even in the broad sense the authors refer to, is weak and scientifically not rigorous.

Second, throughout the manuscript and their replies, the authors consistently treat the two Feugere datasets as equivalent, despite males and females exhibiting radically different behaviors.

Third, the authors attempt to justify the weak alignment found in the male Feugere data by pointing to differences in the experimental setup and 3D marker geometry. Again, as for BR1, if these were truly the cause of the model's failure, they would have disrupted the alignment of both males and females, making both datasets incompatible with the Poda data and BR2.

To justify the incompatibility of the male Feugere data with the model, the authors also use the argument of aiming for a parsimonious sufficiency model, rather than a predictive one. However, I find this defence conceptually weak. Even a sufficiency model must demonstrate that its minimal rules are robust enough to explain the phenomenon under investigation. If a biological factor has a dominant effect in one dataset and is negligible in another, it is not the driving mechanism of the phenomenon. Consequently, the model cannot be generalized to wider swarming behavior - such as the mating swarms in nature mentioned in lines 730–774 - and the model loses its biological relevance.

Reviewer #3: I believe that the authors did a great job replying to all comments and revising the manuscript. I think it is a strong piece of work that perfectly fits in the journal. As the authors note, I believe that this bottom-up approach with a model based on empirical data is an important contribution to our mechanistic understanding of this system, without proving the predictive power of the model. If future empirical work provides a solid comparison between swarms across a range of experimental set ups in terms of marker characteristics, there will be good insights to then expand the model and gain a more universal understanding in future work. The revised manuscript is very clear concerning the conditions from which it draws conclusions and doesn't overstate its findings.

**********

**Have the authors made all data and (if applicable) computational code underlying the findings in their manuscript fully available?**

The PLOS Data policy requires authors to make all data and code underlying the findings described in their manuscript fully available without restriction, with rare exception (please refer to the Data Availability Statement in the manuscript PDF file). The data and code should be provided as part of the manuscript or its supporting information, or deposited to a public repository. For example, in addition to summary statistics, the data points behind means, medians and variance measures should be available. If there are restrictions on publicly sharing data or code —e.g. participant privacy or use of data from a third party—those must be specified.

Reviewer #1: Yes

Reviewer #2: Yes

Reviewer #3: Yes

**********

PLOS authors have the option to publish the peer review history of their article (what does this mean?). If published, this will include your full peer review and any attached files.

If you choose “no”, your identity will remain anonymous but your review may still be made public.

**Do you want your identity to be public for this peer review?**  For information about this choice, including consent withdrawal, please see our Privacy Policy.

Reviewer #1: No

Reviewer #2: No

Reviewer #3: No

# Editor report: Acceptance letter

**DOI:** 10.1371/journal.pcbi.1014685.r008

**Authors:** Tobias Bollenbach

PCOMPBIOL-D-25-00797R3

The complex swarming dynamics of malaria mosquitoes emerge from simple minimally-interactive behavioral rules

Dear Dr Cribellier,

I am pleased to inform you that your manuscript has been formally accepted for publication in PLOS Computational Biology. Your manuscript is now with our production department and you will be notified of the publication date in due course.

The corresponding author will soon be receiving a typeset proof for review, to ensure errors have not been introduced during production. Please review the PDF proof of your manuscript carefully, as this is the last chance to correct any errors. Please note that major changes, or those which affect the scientific understanding of the work, will likely cause delays to the publication date of your manuscript.

Soon after your final files are uploaded, unless you have opted out, the early version of your manuscript will be published online. The date of the early version will be your article's publication date. The final article will be published to the same URL, and all versions of the paper will be accessible to readers.

For Research, Software, and Methods articles, you will receive an invoice from PLOS for your publication fee after your manuscript has reached the completed accept phase. If you receive an email requesting payment before acceptance or for any other service, this may be a phishing scheme. Learn how to identify phishing emails and protect your accounts at https://explore.plos.org/phishing.

Thank you again for supporting PLOS Computational Biology and open-access publishing. We are looking forward to publishing your work!

With kind regards,

Sharmila Kamatchi

PLOS Computational Biology | Carlyle House, Carlyle Road, Cambridge CB4 3DN | United Kingdom ploscompbiol@plos.org | Phone +44 (0) 1223-442824 | ploscompbiol.org | @PLOSCompBiol
